# Computational modeling of resistance to hormone-mediated remission in childhood absence epilepsy

**DOI:** 10.3389/fncom.2025.1733650

**Published:** 2026-01-12

**Authors:** Maliha Ahmed, Sue Ann Campbell

**Affiliations:** 1 The Picower Institute for Learning and Memory, Massachusetts Institute of Technology, Cambridge, MA, United States; 2 Department of Applied Mathematics, University of Waterloo, Waterloo, ON, Canada; 3 Centre for Theoretical Neuroscience, University of Waterloo, Waterloo, ON, Canada

**Keywords:** childhood absence epilepsy, conductance-based model, frontocortical, remission, thalamocortical

## Abstract

Childhood absence epilepsy (CAE) often resolves during adolescence, a period marked by hormonal and neurosteroid changes associated with puberty. However, remission does not occur in all individuals. To investigate this clinical heterogeneity, we developed a simplified thalamocortical model with a layered cortical structure, using deep-layer intrinsically bursting (IB) neurons to represent frontal cortex and regular spiking (RS) neurons modeling the parietal cortex. By simulating two cortical configurations, we explored how variations in neuronal composition and frontocortical connectivity influence seizure dynamics and the effectiveness of allopregnanolone (ALLO) in resolving pathological spike-wave discharges (SWDs) associated with CAE. While both models exhibited similar physiological and pathological oscillations, only the parietal-dominant network (with a higher proportion of RS neurons in layer 5) recovered from SWDs under increased frontocortical connectivity following ALLO administration. These findings suggest that neuronal composition critically modulates ALLO-mediated resolution of SWDs, providing a mechanistic link between structural connectivity and clinical outcomes in CAE, and highlighting the potential for personalized treatment strategies based on underlying network architecture.

## 1 Introduction

Childhood absence epilepsy is a common pediatric epilepsy disorder, accounting for approximately 18% of all childhood epilepsies, with onset typically occurring between the ages of 4 and 12 years (Hirsch et al., 2022). Characterized by brief episodes of impaired consciousness, typical absence seizures present with bilateral synchronous 2.5–5 Hz SWDs on electroencephalography (EEG) (Hirsch et al., 2022). While antiepileptic drugs are effective for most patients, with approximately 70% achieving remission, some cases progress to more severe forms of epilepsy (Kessler and McGinnis, 2019). Interestingly, circumstantial evidence regarding the prognosis of untreated epilepsy suggests spontaneous remission rates between 31%–42%, although these figures are not specific to childhood-onset epilepsy syndromes (Kwan and Sander, 2004; Beghi et al., 2015). Several factors, including the use of antiepileptic drugs, genetic predisposition, and intrinsic connectivity differences, are hypothesized to influence remission; however, understanding of these factors remains limited due to their considerable variability among patients.

The thalamocortical circuit—comprising pyramidal neurons in the cortex, thalamic relay neurons, and thalamic reticular neurons—plays a critical role in both normal 7–10 Hz sleep spindle generation and pathological SWD activity (Sitnikova et al., 2014). Evidence indicates that SWDs may originate from slower oscillations within focal excitable regions, particularly the somatosensory cortex, before being sustained by circuits in the frontal cortex and thalamus (Meeren et al., 2002; Masterton et al., 2013). Notably, children with newly diagnosed CAE who do not respond to treatment demonstrate increased frontocortical connectivity prior to treatment initiation, suggesting fundamental differences in network architecture may influence disease trajectory (Tenney et al., 2018).

Neuronal electrophysiology and intrinsic membrane properties vary significantly based on neuron morphology, cortical layer, and cortical region. Pyramidal neurons in the cortex typically exhibit either regular spiking or intrinsically bursting behavior. IB neurons tend to have thick apical dendrites as compared to RS neurons, as shown in studies of pyramidal neurons in layers 2/3 and 5 of rat visual cortex (Mason and Larkman, 1990). In slices of sensorimotor and frontal cortex from guinea pigs, RS pyramidals were found in all layers below layer 1, while IB cells were located mostly in layers 4/5 and deeper (McCormick et al., 1985). Additionally, studies in Sprague-Dawley rats indicate a higher percentage (approximately 73%) of layer 5/6 IB neurons in slices of prefrontal cortex, compared to layer 5 cells in slices of somatosensory (parietal) cortex (approximately 50%–60%) (Chagnac-Amitai et al., 1990; Yang et al., 1996). These regional differences in neuronal composition may contribute to the pathophysiological variability observed in CAE.

Given that remission in most cases occurs during adolescence, the association of sex steroid hormones with CAE is suspected and additionally supported by the phenomenon of catamenial seizure exacerbation (van Luijtelaar et al., 2001). Progesterone is one of the key steroid hormones that plays a crucial role in sexual development during puberty. While it is primarily regarded as a female hormone, its significance in males is also recognized (Ghoumari et al., 2014; Guennoun, 2020). One of the metabolites produced during progesterone metabolism is allopregnanolone. Both progesterone and ALLO are neurosteroids which can be synthesized within the nervous system or accumulate in the brain via systemic circulation after being derived by the gonads (in females) and by the adrenal glands (in both sexes) (Ghoumari et al., 2014). There is considerable evidence from both clinical studies and animal experiments regarding the effect of progesterone (and ALLO) on human and animal EEG, particularly in exacerbating pathological activity (Grünewald et al., 1992; Budziszewska et al., 1999; van Luijtelaar et al., 2001). ALLO, a neuroactive steroid, has the ability to modulate neuronal excitability through both genomic (i.e., through regulating gene expression) and nongenomic means (i.e., modifying ion conductance, second messengers, and activating signaling pathways) (Zamora-Sánchez and Camacho-Arroyo, 2022). At the nongenomic level, its effect on GABA_A_ receptors depends on its concentration. At nanomolar concentrations, ALLO is a positive allosteric modulator of the GABA_A_ receptor (Pinna et al., 2000; Puia et al., 2003; Lambert et al., 2009). Specifically, it increases the probability of the channel being in the open state and enhances the receptor's response to GABA by increasing its efficacy (Bromfield et al., 2006; Majewska et al., 1986). At micromolar concentrations, ALLO can activate GABA_A_ receptors independently of GABA (Liu et al., 2002; Lambert et al., 2009). Its overall effect is to potentiate GABA action particularly by decreasing the rise time and increasing the decay time of the evoked current, an effect consistently observed across various experimental conditions (Majewska et al., 1986; Paul and Purdy, 1992; Sullivan and Moenter, 2003; Strömberg et al., 2006; Lu et al., 2023). Additionally, under some conditions, an increase in current amplitude is also observed (Paul and Purdy, 1992; Sullivan and Moenter, 2003; Majewska et al., 1986).

In our recent work using a conductance-based thalamocortical model, we found that ALLO's modulation of GABA_A_-receptor mediated inhibition had an ameliorating effect on SWDs (Ahmed and Campbell, 2024). This finding appears to contradict established evidence but may reflect limitations in existing experimental models, which do not capture the naturally remitting course typical of most CAE cases (Grünewald et al., 1992; van Luijtelaar et al., 2001). The thalamocortical model employed in our previous study featured a well-defined thalamic component based on Destexhe's work, but was limited by its lack of a comprehensive cortical representation (Destexhe et al., 1998). The cortical cells in this model incorporated only basic currents (*Na*^+^, *K*^+^, and an additional slow-*K*^+^ current in pyramidal cells) and lacked region-specific or laminar organization.

In this study, we develop an enhanced minimal conductance-based thalamocortical model with an improved cortical component. Our revised model incorporates a layered cortical structure featuring region-specific pyramidal cells and deep-layer interneurons from layers 5 and 6. These modifications create a more physiologically relevant thalamocortical network while maintaining computational efficiency. We use this model to investigate how variations in cortical neuronal composition influence seizure dynamics, with a particular focus on ALLO as a neuromodulatory agent in the resolution of SWDs. Specifically, we examine how different ratios of IB to RS neurons in layer 5 affect the generation, propagation, and maintenance of SWDs. Furthermore, we explore the impact of enhanced frontocortical connectivity on these different cortical configurations. Our analysis aims to identify distinct network features that may distinguish CAE networks in which SWDs are resolved by ALLO from those in which such neurosteroid modulation is ineffective. This computational approach offers a valuable means to explore factors underlying divergent disease trajectories, addressing a key gap in understanding due to the lack of a remitting animal model for CAE.

## 2 Materials and methods

### 2.1 Model description

Our 475-cell thalamocortical network consists of single-compartment neuron models of the following types: layer 5 regular spiking (RS), layer 5 intrinsically bursting (IB), layer 6 non-tufted regular spiking (NRS) pyramidals and a deep low-threshold spiking (LTS) interneuron in the cortex, and thalamic reticular cells (RE) and thalamocortical cells (TC) in the thalamus. The membrane potential of each cell type is described by the following equations:


$$\begin{array}{l} C_{m1}\frac{dV_{RS}}{dt}=I_{hold}-I_{Leak}-I_{Naf}-I_{Nap}-I_{Ka}-I_{K2}-I_{Kdr}\\ \;-I_{Km}-I_{Kc}-I_{Kahp}-I_{CaT}-I_{CaL}-I_{h}-I_{GABAA_{LTS}}\\ \;-I_{GABAB_{LTS}}-I_{AMPA_{RS}}-I_{AMPA_{IB}}-I_{AMPA_{NRS}}\\ \;-I_{AMPA_{TC}}, \end{array}$$ (1)

$$\begin{array}{l} C_{m1}\frac{dV_{IB}}{dt}=I_{hold}-I_{Leak}-I_{Naf}-I_{Nap}-I_{Ka}-I_{K2}-I_{Kdr}\\ \;-I_{Km}-I_{Kc}-I_{Kahp}-I_{CaT}-I_{CaL}-I_{h}-I_{GABAA_{LTS}}\\ \;-I_{GABAB_{LTS}}-I_{AMPA_{RS}}-I_{AMPA_{IB}}-I_{AMPA_{NRS}}\\ \;-I_{AMPA_{TC}}, \end{array}$$ (2)

$$\begin{array}{l} C_{m1}\frac{dV_{NRS}}{dt}=I_{hold}-I_{Leak}-I_{Naf}-I_{Nap}-I_{Ka}-I_{K2}-I_{Kdr}\\ \;-I_{Km}-I_{Kc}-I_{Kahp}-I_{CaT}-I_{CaL}-I_{h}-I_{GABAA_{LTS}}\\ \;-I_{GABAB_{LTS}}-I_{AMPA_{RS}}-I_{AMPA_{IB}}-I_{AMPA_{NRS}}\\ \;-I_{AMPA_{TC}}, \end{array}$$ (3)

$$\begin{array}{l} C_{m2}\frac{dV_{LTS}}{dt}=I_{hold}-I_{Leak}-I_{Naf}-I_{Nap}-I_{Ka}-I_{K2}-I_{Kdr}\\ \;-I_{Km}-I_{Kc}-I_{Kahp}-I_{CaT}-I_{CaL}-I_{h}-I_{AMPA_{RS}}\\ \;-I_{AMPA_{IB}}-I_{AMPA_{NRS}}-I_{AMPA_{TC}}, \end{array}$$ (4)

$$\begin{array}{l} C_{m2}\frac{dV_{TC}}{dt}=-I_{Leak}-I_{KLeak}-I_{Naf}-I_{K}-I_{CaT}-I_{h}\\ \;-I_{GABAA_{RE}}-I_{GABAB_{RE}}-I_{AMPA_{NRS}}, \end{array}$$ (5)

$$\begin{array}{l} C_{m2}\frac{dV_{RE}}{dt}=-I_{Leak}-I_{Naf}-I_{K}-I_{CaT}-I_{GABAA_{RE}}-I_{AMPA_{NRS}}\\ \;-I_{AMPA_{TC}}, \end{array}$$ (6)

where *V*_*i*_ is the membrane potential for *i* = RS, IB, NRS, LTS, TC, RE, *C*_*m*1_ = 0.9 and *C*_*m*2_ = 1.0 is the specific membrane capacitance given in μF/cm^2^. *I*_*hold*_ is the input current required to hold and maintain the voltage of the neuron (also used in this context for setting the resting membrane potential). A consistent set of units were maintained such that voltage is given in mV, ionic currents in mA/cm^2^, membrane conductance densities in S/cm^2^, and time in msec. Note that while input currents are given in nA, they are converted to a current density by dividing by the cell compartment's area.

All cortical cells have the same set of ionic currents with different kinetics in some instances. These consist of both transient and persistent sodium currents, potassium currents of the delayed rectifier, transient, slowly inactivating, and *Ca*^2+^-dependent types, in addition to both low- and high-threshold *Ca*^2+^ currents. The models for these cells were adapted from our previous work (Ahmed, 2019), which modified single-neuron models from Traub et al. (2005). The original model by Traub et al. featured multi-compartment neurons in a large-scale network, successfully replicating various thalamocortical phenomena. In our work, we simplified these complex neuronal models to single-compartment representations of the soma while preserving essential dynamics. This reduction required careful recalibration of membrane conductance densities to maintain physiologically realistic behavior. In contrast, the thalamic cells were based on the model by Destexhe et al. (1998), which describes thalamic neurons with fewer currents but still exhibits bursting behavior due to the presence of the low-threshold calcium current, with slower kinetics in the RE cells. All intrinsic currents follow the same formalism, described by the following equation:


$$\begin{array}{l} I_{ion}={ḡ}_{ion}\cdot m^{N}h^{M}\cdot \left(V_{m}-E_{ion}\right), \end{array}$$ (7)

where ḡ_*ion*_ represents the maximal conductance, and *m* and *h* represent the voltage gating of the ion channels. All synaptic currents are described by the following equations:


$$\begin{array}{l} I_{syn}={ḡ}_{syn}\cdot s\left(t\right)\cdot \left(V_{m}-E_{syn}\right), \end{array}$$ (8)

$$s(t)=\{\begin{array}{l} s_{\infty }+(s(t_{0})-s_{\infty })e^{-\frac{t}{\tau_{s}}},\;t_{0}\leq t<C_{dur}\\ {}[s_{\infty }+(s(t_{0})-s_{\infty })e^{-\frac{C_{dur}}{\tau_{s}}}]e^{-\beta (t-C_{dur})},\;t\geq C_{dur}, \end{array}$$ (9)

$$\begin{array}{l} s_{\infty }=\frac{\alpha C_{max}}{\alpha C_{max}+\beta }, \end{array}$$ (10)

$$\begin{array}{l} \tau_{s}=\frac{1}{\alpha C_{max}+\beta }, \end{array}$$ (11)

where ḡ_*syn*_ denotes the maximal synaptic conductance, and AMPA/GABA_A_- and GABA_B_- mediated synapses are described appropriately by the synaptic conductance function, *s*(*t*) which denotes the fraction of open synaptic channels at time *t*. The time of a presynaptic spike arrival is denoted by *t*_0_, while α and β denote the forward (binding) and backward (unbinding) rate constants describing the binding of neurotransmitter, respectively. The duration of the neurotransmitter-mediated pulse is given by *C*_*dur*_, and *C*_*max*_ refers to the maximal neurotransmitter concentration value. The details of the individual currents, synaptic dynamics, and model parameters are provided in Supplementary Sections 1, 2.

**Figure 1:**
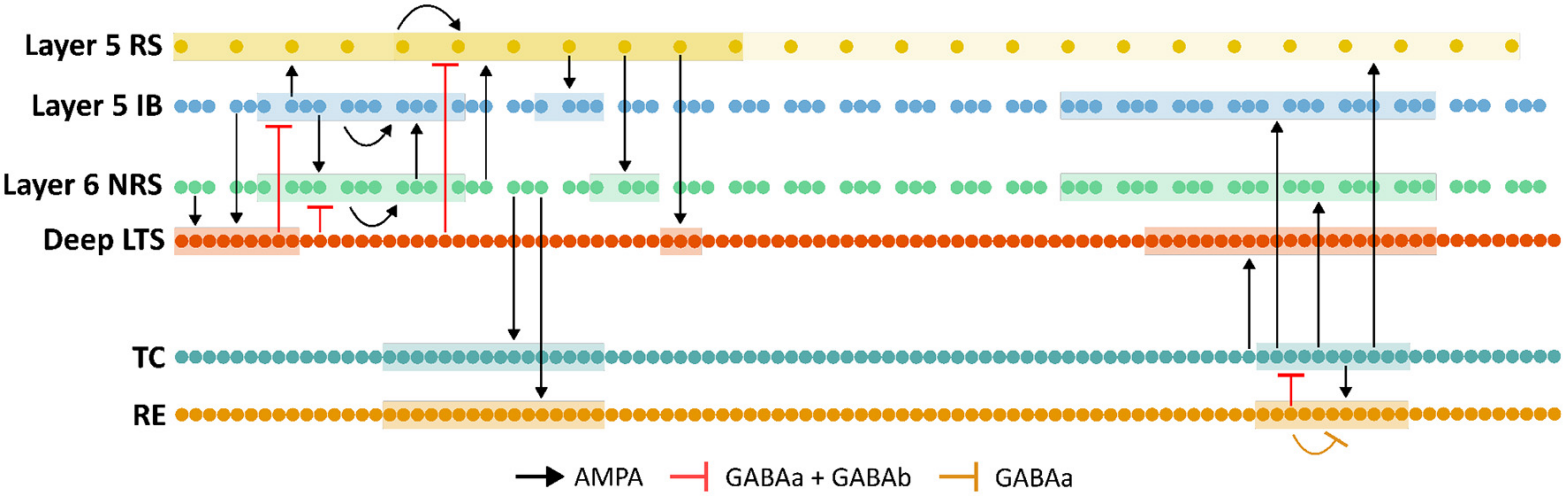
Schematic representation of the thalamocortical network. Each neuron is represented by a dot with synaptic connectivity within and between layers as shown. The number of postsynaptic neurons each neuron connects to varies by layer and is represented by the highlighted groups as shown.

The network consists of the following cell populations: 25 layer 5 RS pyramidals, 75 layer 5 IB pyramidals, 75 layer 6 non-tufted RS pyramidals, 100 deep LTS interneurons, 100 thalamocortical cells, and 100 thalamic reticular cells. At baseline, a higher proportion of bursting-type pyramidal cells compared to regular-spiking cells in layer 5 was used to both introduce asymmetry in this layer, as well as model the frontal cortex. All cell layers were of equal size, except for layer 6, which had a reduced population size relative to layer 5 to reflect its typically lower density of pyramidal cells (DeFelipe et al., 2002). Each layer of cells is arranged in one dimension as shown in Figure 1 with synaptic connectivity varying between cell types as described. Cortical input into the thalamus is only from layer 6 of the cortex with excitatory synapses formed with ascending thalamic axons. All excitatory connections in the network are mediated by AMPA receptors, and inhibitory connections are mediated by either GABA_A_ only or a combination of GABA_A_ and GABA_B_ receptors.

Due to the unequal number of cells in some layers, the number of presynaptic neurons each postsynaptic neuron connects to (*nPrePost*) was layer-dependent, as given in Table 1. In each network layer, presynaptic cells were indexed relative to the indexing of postsynaptic cells. Within the thalamus and the cortex, each neuron connects to postsynaptic neurons within a radius of 11 indices, while between the thalamus and cortex, each presynaptic neuron connects with neurons within a radius of 21 postsynaptic indices. This cell count affects the weight of individual synapses which is defined by ḡ_*syn*_/*nPrePost* where ḡ_*syn*_ is the maximal synaptic conductance for a particular synapse, as given in Table 2.

**Table 1:** The number of presynaptic neurons that each postsynaptic neuron connects to (*nPrePost*) in a network with the following architecture: 25 layer 5 RS cells, 75 layer 5 IB cells, 75 layer 6 NRS cells, 100 deep layer LTS cells, 100 TC cells, and 100 RE cells.

***nPrePost***	**Number of cells**	***nPrePost***	**Number of cells**
nRSRS	11	nLTSRS	11
nRSIB	4	nLTSIB	11
nRSNRS	4	nLTSNRS	11
nRSLTS	3	nTCRE	11
nIBIB	11	nTCNRS	21
nIBRS	11	nTCIB	21
nIBNRS	11	nTCRS	21
nIBLTS	9	nTCLTS	21
nNRSNRS	11	nRERE	11
nNRSRS	11	nRETC	11
nNRSIB	11
nNRSLTS	9
nNRSTC	16
nNRSRE	16

For example, nRSIB = 4 means that each IB cell receives input from 4 RS cells.

**Table 2:** Maximal synaptic conductance, ḡ_*syn*_ parameters (in μS), for each synapse type, chosen to produce a default network state exhibiting spindle oscillations.

	**Post-synaptic neuron type**					
**Pre-synaptic neuron type**	**RS**	**IB**	**NRS**	**LTS**	**TC**	**RE**
RS	0.3	0.7	0.3	0.1	-	-
IB	1	0.3	0.05	0.08	-	-
NRS	0.1	0.3	2	0.2	0.02	2.4
LTS	0.09 (GABA_A_)	0.1 (GABA_A_)	0.75 (GABA_A_)	-	-	-
	0.03 (GABA_B_)	0.03 (GABA_B_)	0.03 (GABA_B_)			
TC	1	0.7	1.2	0.4	-	0.2
RE	-	-	-	-	0.02 (GABA_A_)	0.2
					0.04 (GABA_B_)	

Values for GABA_A_ synapses reflect conductance under Control receptor activity.

After allowing the network to reach steady state, a 1 nA stimulus was applied for 100 ms to a group of five layer 6 NRS neurons, inducing additional oscillations. Network behavior and states were characterized using these triggered oscillations. The model fitting parameters, given in Tables 2–4 were selected based on the method described in Supplementary Section 3.1, and were intended to be interpreted qualitatively. The same set of fitted parameters was used to model both the default and diseased states. In the default mode, the network exhibits spontaneous oscillations driven by spontaneous discharges in TC neurons, while in the diseased mode, the model exhibits synchronized SWDs due to alterations in the strength of cortical inhibition.

**Table 3:** Forward (binding) rate, α parameters (in (ms·mM)^−1^), for each synapse type, chosen to produce a default network state exhibiting spindle oscillations.

	**Post-synaptic neuron type**					
**Pre-synaptic neuron type**	**RS**	**IB**	**NRS**	**LTS**	**TC**	**RE**
RS	0.05	0.05	0.1	0.5	-	-
IB	0.1	0.1	0.1	0.05	-	-
NRS	0.05	0.1	0.05	0.625	0.75	0.8
LTS	0.1	0.05	0.1	-	-	-
TC	1	1	0.07	0.149	-	0.94
RE	-	-	-	-	20	20

Note that α values for inhibitory synapses shown here correspond only to GABA_A_ synapses and reflect binding rates under Control receptor activity.

**Table 4:** Backward (unbinding) rate, β parameters (in ms^−1^) for each synapse type, chosen to produce a default network state exhibiting spindle oscillations.

	**Post-synaptic neuron type**					
**Pre-synaptic neuron type**	**RS**	**IB**	**NRS**	**LTS**	**TC**	**RE**
RS	0.15	0.05	0.01	0.2	-	-
IB	0.15	0.05	0.01	0.08	-	-
NRS	0.05	0.01	0.03	0.109	0.3	0.09
LTS	0.2	0.7	0.01	-	-	-
TC	0.15	0.15	0.006	0.048	-	0.18
RE	-	-	-	-	0.162	0.162

Note that β values for inhibitory synapses presented here correspond only to GABA_A_ synapses and reflect unbinding rates under Control receptor activity.

### 2.2 Tools for analysis

The model was implemented in NetPyNE, a Python package developed by Dura-Bernal et al. that allows for high-level specification of network connectivity of biological neuronal networks and simulations using the NEURON simulator (Dura-Bernal et al., 2019).

#### 2.2.1 Local field potential and spectral density analysis

The local field potential (LFP) was calculated using a point current source model in which current sources are treated as if they originate from a single point. Due to memory limitations, only a subset of the layer 5 RS and IB populations was recorded from and used in the LFP calculation. For each population, neurons are arranged in a single line 20 μm apart. The extracellular recording site is considered to be 50 μm opposite to the center of the line, and the LFP at this site was calculated from postsynaptic currents using the following equation:


$$\begin{array}{l} V_{ext}=\frac{R_{e}}{4\pi }\underset{i}{\sum }\frac{I_{syn}}{r_{i}}, \end{array}$$ (12)

where *V*_*ext*_ is the potential at a defined extracellular site, *R*_*e*_ = 230 Ωcm is the extracellular resistivity, *I*_*syn*_ is the postsynaptic current, and *r*_*i*_ is the distance between the location of the postsynaptic currents and the extracellular site. The LFP traces were first detrended using the *SciPy* Python library to eliminate very low-frequency components that could contaminate the power spectrum and cause spectral leakage. The traces were then filtered using a Butterworth bandpass filter, with cutoff frequencies set at 1 Hz and 15 Hz. Power spectral density (PSD) analysis was performed on the filtered LFP traces using Welch's method with a sampling frequency (*fs)* of 10 kHz. A Hann window was applied to segments of 20,000 samples, with 1,000 samples of overlap between consecutive segments.

### 2.3 Modeling the effect of genetic mutations on GABA_A_ current

Mutations in genes encoding GABA_A_ receptor subunits, including the *GABRG*2 gene, have been implicated in CAE (Kananura et al., 2002; Kang and Macdonald, 2004; Ito et al., 2005; Marini et al., 2003). Functional studies of mutant receptors, particularly those containing the γ2(R82Q) and γ2(R43Q) subunits, have demonstrated reduced receptor surface expression in cortical pyramidal neurons and decreased GABA_A_ receptor current amplitudes (Kang and Macdonald, 2004; Macdonald et al., 2010). These alterations are consistent with a reduction in neuronal feedforward inhibition within cortical circuits (Bianchi et al., 2002; Currie et al., 2017). In our model, the effects of the *GABRG*2 mutation were implemented specifically in cortical pyramidal neurons across both layers by modifying the parameter corresponding to the maximum conductance of the GABA_A_ current (ḡ_*max*_), as outlined below:


$$\begin{array}{l} I_{GABAA}={ḡ}_{max}\cdot s\left(t\right)\cdot \left(V_{m}-E_{GABA}\right). \end{array}$$ (13)

### 2.4 Modeling the effect of allopregnanolone

The effect of ALLO was modeled at the level of synapses, particularly GABA_A_ receptor-mediated synapses, by adjusting the ḡ_*max*_, α, β, and *C*_*dur*_ parameters, as highlighted in Equations 9–10, 13.

Modifications to the highlighted parameters were informed by fitting our synapse model to experimental data, using the relative change between control GABA_A_ receptor activity and activity after ALLO application. It is important to note that the available experimental data vary considerably in methodology across studies, including differences in the species of central nervous system neurons used, and the concentrations of both GABA (0.005 to 0.1 mM) and ALLO (30 to 1,000 nM). While the physiological concentration of GABA released into the synaptic cleft ranges from 1 to 10 mM, the experimental data used to model the effects of ALLO are based on GABA concentrations that are at least an order of magnitude lower (Brickley and Mody, 2012; Overstreet et al., 2002; Tretter and Moss, 2008; Mozrzymas et al., 2003). Similarly, physiological ALLO levels in rat cortex typically range from 1 to 20 nM, yet experimental protocols generally apply concentrations that are one to two orders of magnitude higher (Pinna et al., 2000; Kelley et al., 2011). The lack of significant differences in response amplitude (i.e., relative change in ḡ_*max*_) following the application of ALLO in studies using low mM concentrations of GABA could perhaps be explained by these variations. Given these discrepancies, our modeling of these effects is meant to be interpreted qualitatively. The details of the curve fits can be found in Supplementary Section 3.2.

While the *C*_*dur*_ parameter corresponding to Control synapses (*C*_*du*_*r*__*Control*__) for each GABA_A_ receptor-mediated synapse was set to 0.3 ms, the values of ḡ_*max*_, α, and β corresponding to Control synapses (i.e., ḡ_*ma*_*x*__*Control*__, α_*Control*_, β_*Control*_) were taken from the values given in Tables 2–4. For post-ALLO synapses, each parameter was modified based on the relative changes between control GABA_A_ receptor activity and activity following the application of ALLO, as follows:


$$\begin{array}{l} \alpha_{post-ALLO}=1.58\times \alpha_{Control}, \end{array}$$ (14)

$$\begin{array}{l} \beta_{post-ALLO}=0.74\times \beta_{Control}, \end{array}$$ (15)

$$\begin{array}{l} {ḡ}_{max_{post-ALLO}}=1.11\times {ḡ}_{max_{Control}}, \end{array}$$ (16)

$$\begin{array}{l} C_{dur_{post-ALLO}}=1.14\times C_{dur_{Control}}. \end{array}$$ (17)

We specifically chose to model the effects of ALLO using synaptic and kinetic parameters fitted at physiological ALLO concentrations. A comparison of these parameters across physiological and supraphysiological ALLO levels is provided in Supplementary Figure S2.

### 2.5 Modeling regional neuronal heterogeneity and enhanced frontocortical connectivity

To model the regional differences in neuronal heterogeneity observed experimentally, as discussed previously, we developed two distinct model configurations. Given that layer 5 IB neurons are more abundant in the frontal cortex and RS neurons in the parietal cortex, we modeled the frontal cortex using IB neurons while the parietal cortex was modeled using RS neurons. We chose this binary framework because it provides a manageable level of model complexity while still allowing us to probe specific mechanistic questions. Accordingly, we varied their proportions in each model such that the 95-5 (nIB:nRS) configuration is representative of a cortex most influenced by the frontal cortex, while the 5-95 (nIB:nRS) configuration reflects a cortical component that is most influenced by the parietal cortex. Given the differences in model architecture, the number of presynaptic neurons each postsynaptic neuron connects to (*nPrePost*) differs between the two model configurations, as presented in Supplementary Tables S3, S4.

**Figure 2:**
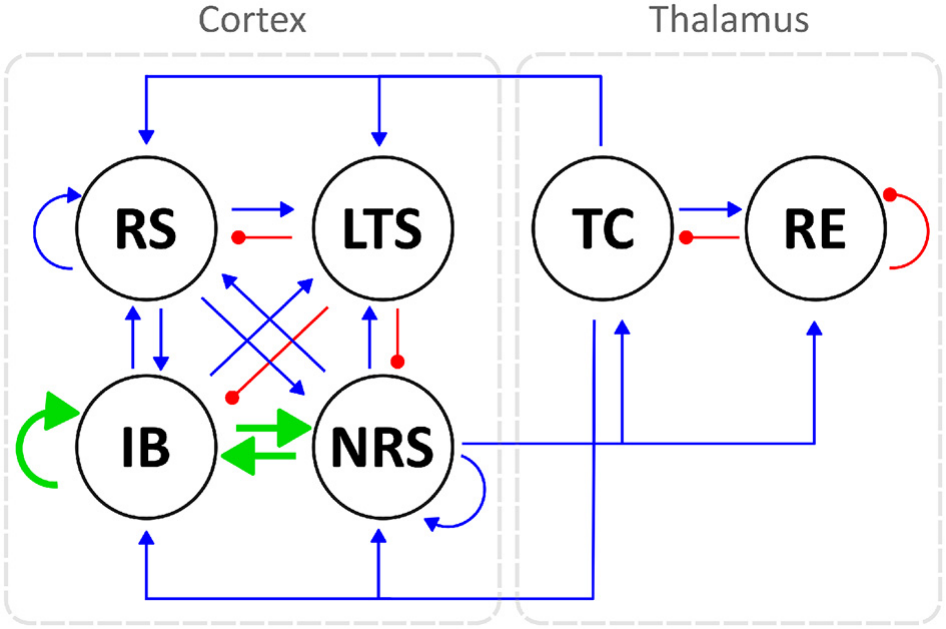
A schematic diagram of the thalamocortical circuit shows connections between cortical neurons of the RS, IB, NRS, and LTS type, and thalamic neurons (TC and RE). Blue arrows indicate excitatory synaptic connections, while red arrows represent inhibitory ones. Enhanced frontocortical connectivity is illustrated with thicker green arrows, which also represent excitatory synapses.

Additionally, for each model configuration, we modeled enhanced connectivity in the frontal cortex by modifying the strength of the following synapses, as illustrated in Figure 2: IBIB, IBNRS, and NRSIB. Specifically, we modified the maximal synaptic conductances as follows: ḡ_*IBIB*_ was increased by a factor 7, ḡ_*IBNRS*_ by a factor of 5, and ḡ_*NRSIB*_ by a factor of 3. These specific adjustments were chosen for demonstration purposes, but similar results were observed across a physiologically reasonable range of synaptic strengths, as shown in Supplementary Figure S4. A detailed description of the method used to determine these parameter adjustments is provided in Supplementary Figure S4.

## 3 Results

### 3.1 Baseline state characterization and allopregnanolone effects on the fitted model

**Figure 3:**
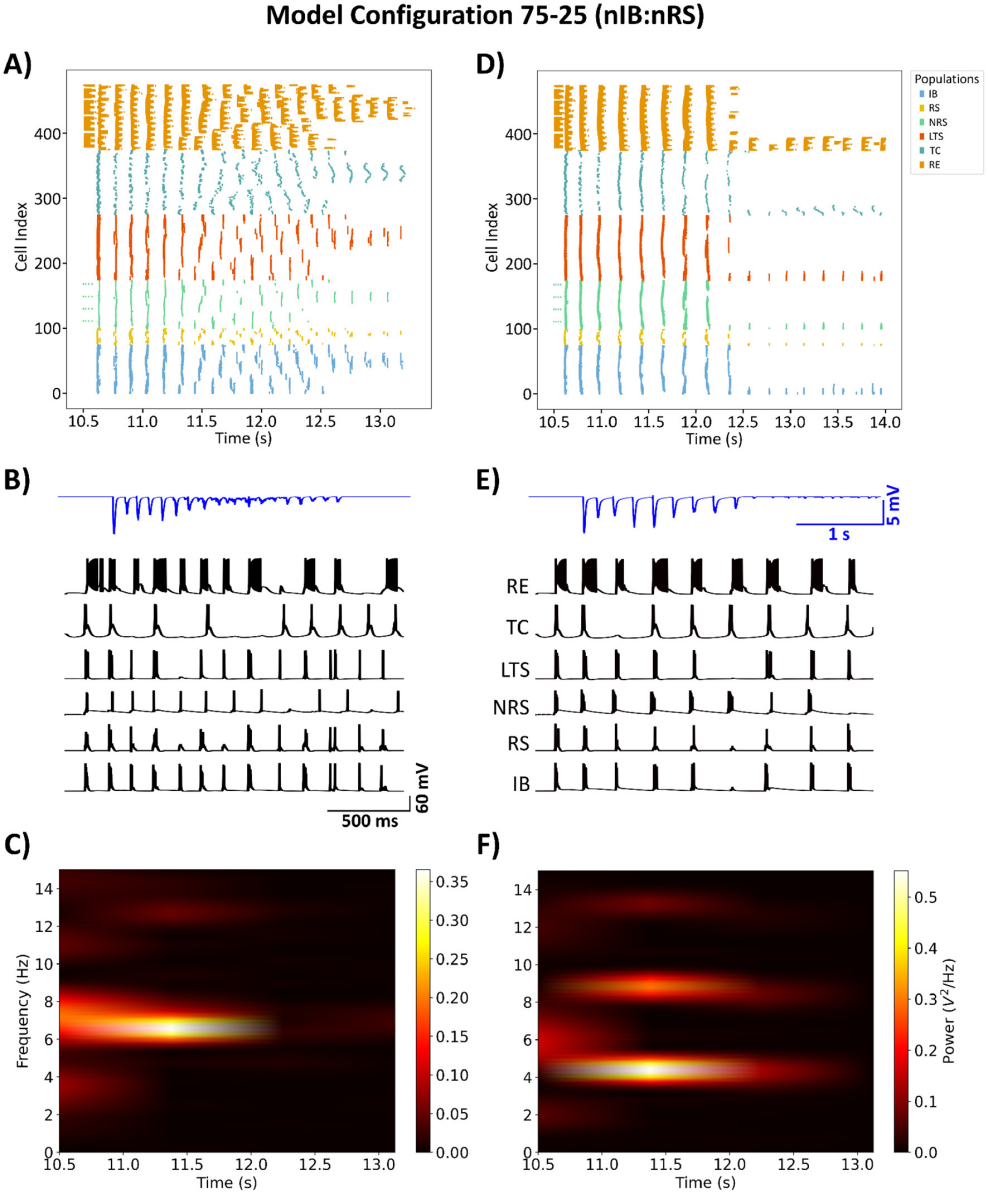
Raster plot of the 72-25 (nIB:nRS) fitted model demonstrates spindle oscillations **(A)**, transitioning to SWDs **(D)** through cortical disinhibition, modeled as a reduction in baseline GABA_A_ conductance from 100% to 10%. **(B, E)** Sample voltage traces from cell index 0 in each population, with LFP traces (in blue) computed using the method outlined in Section 2.2. The network activity during spindle oscillations exhibits a peak power at 7 Hz **(C)**, whereas the SWD state shows peak power at 5 Hz **(F)**. All simulations were conducted using Control GABA_A_-receptor mediated synapses.

**Figure 4:**
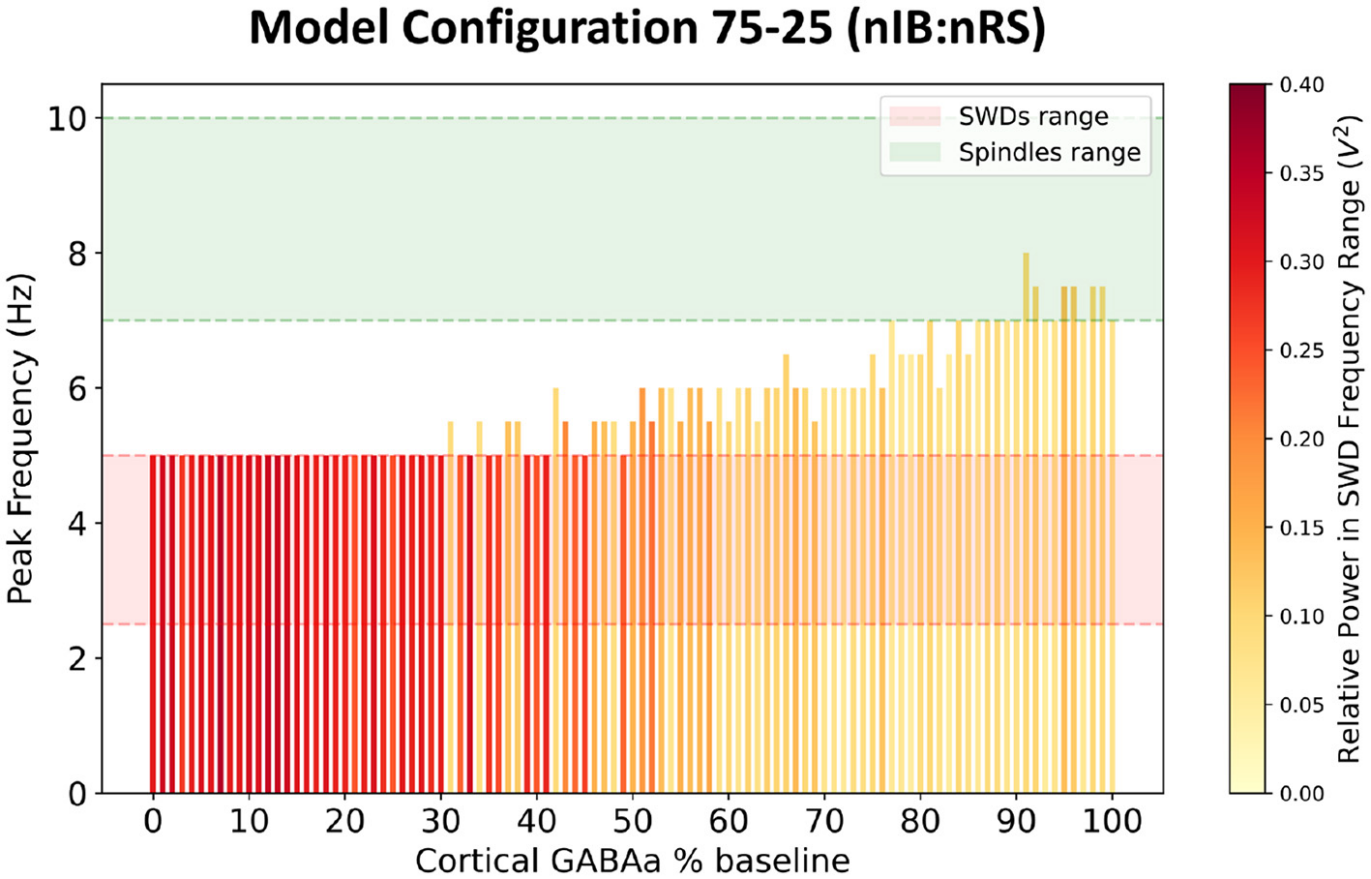
Network frequency response to varying levels of cortical GABA_A_-receptor mediated inhibition. The color of each bar represents the relative power within the frequency range associated with SWDs.

The goal of our model fitting was to replicate behavior that is qualitatively consistent with that of prior established models. Specifically, the model needed to exhibit two critical baseline states: a healthy state characterized by spindle oscillations with a network frequency of 7–10 Hz, and a diseased state marked by SWDs with a network frequency of 2.5–5 Hz. The 75-25 (nIB:nRS) model was simulated using GABA_A_ synapses that were all of the Control type, as described in Table 2. At 100% baseline cortical GABA_A_ conductance, the network produces spindle oscillations with a peak network frequency of 7 Hz, as shown in Figures 3A, C. In this healthy state, cell populations exhibit organized, alternating bursting patterns—particularly within the TC population—with moderately synchronous activity across other populations, as shown in Figure 3B. This activity is reflected in the LFP trace as a low-amplitude rhythmic oscillation. The network could be transformed to a diseased state by means of cortical disinhibition. By reducing GABA_A_-receptor mediated inhibition in the cortex to 10% baseline conductance, the network produces a dominant 5 Hz SWD pattern as shown in Figures 3D, F. In this diseased state, neural firing becomes more synchronous across all populations, with distinctive spike-wave complexes visible in the LFP, as shown in Figure 3E. The reduction in cortical GABA_A_ conductance was determined by examining how changes in conductance influence network frequency and the relative power in the SWD frequency range, as shown in Figure 4.

**Figure 5:**
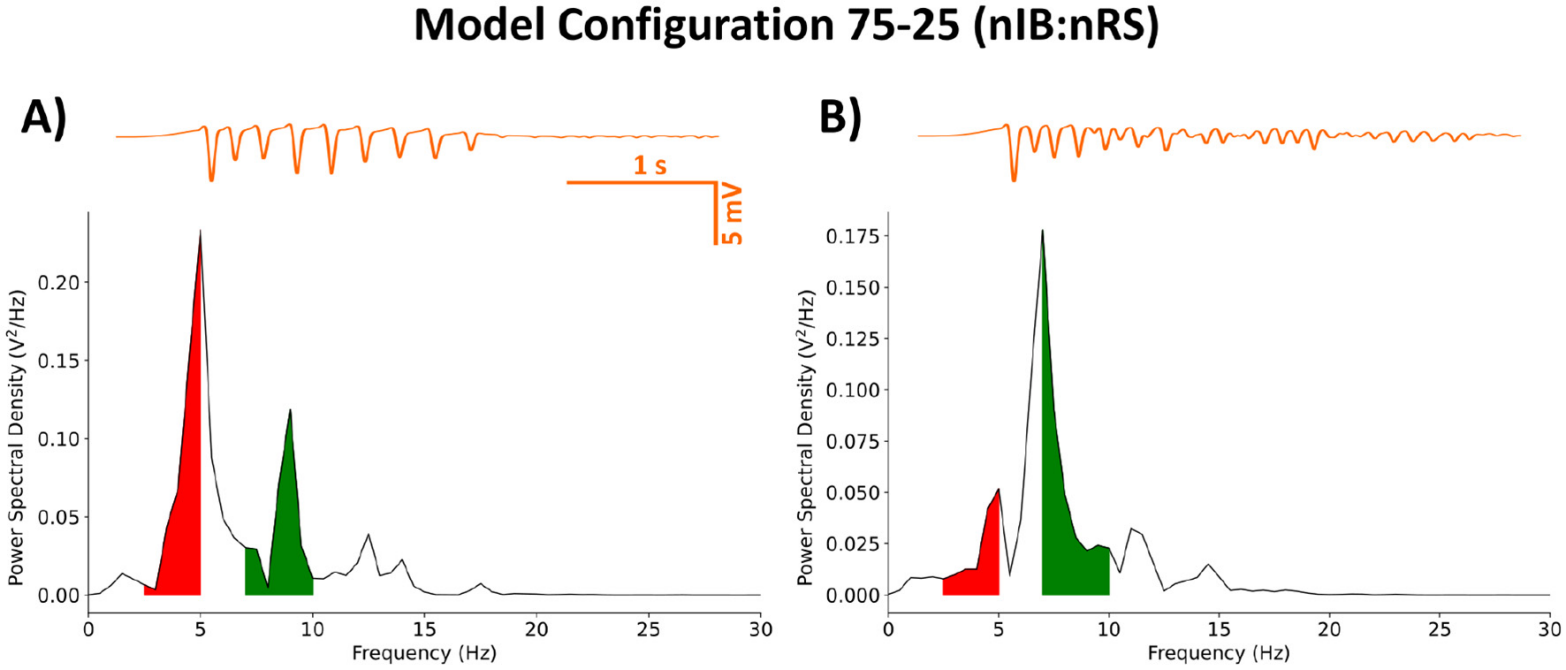
Power spectral density of the 75-25 (nIB:nRS) fitted model using Control synapses **(A)** and post-ALLO synapses **(B)**, calculated from filtered LFP traces (in orange), as described in Section 2.2. The network was initialized with cortical disinhibition (10% baseline cortical GABA_A_ conductance). Shaded regions indicate relative power within the frequency ranges associated with SWDs (2.5–5 Hz; red) or Spindle oscillations (7–10 Hz; green).

Next, we modeled the effect of ALLO by altering parameters associated with the GABA_A_ current described in Section 2.4. The network was initialized to exhibit SWDs, as in Figures 3D–F, with cortical GABA_A_ conductance reduced to 10% baseline. The effect of ALLO was implemented uniformly across all GABA_A_ synapses in the model. Our results show that ALLO effectively suppresses pathological SWDs by altering the temporal firing patterns of neural populations throughout the thalamocortical circuit. As shown in Figure 5, there is a shift in the dominant network frequency to 7 Hz, which falls within the frequency band associated with normal spindle oscillations. This transition is also characterized by alternating bursts of synchrony across populations which is reflected in the low-amplitude filtered LFP trace. The shift from pathological to physiological dynamics is consistent with our previous findings and supports the therapeutic potential of positive GABA_A_ modulators in absence epilepsy.

### 3.2 Regional neuronal heterogeneity in the cortex does not alter ALLO-mediated network dynamics

Using the previously described frontal cortex composition (95-5 nIB:nRS) and parietal cortex composition (5-95 nIB:nRS) in Section 2.5, we next examined whether regional neuronal heterogeneity affects the network's propensity to generate the two critical network states of interest: healthy spindles and pathological SWDs. Additionally, we investigated whether the effect of ALLO differs across networks with different cortical compositions, and importantly, whether one network composition is more prone to non-resolution than the other.

**Figure 6:**
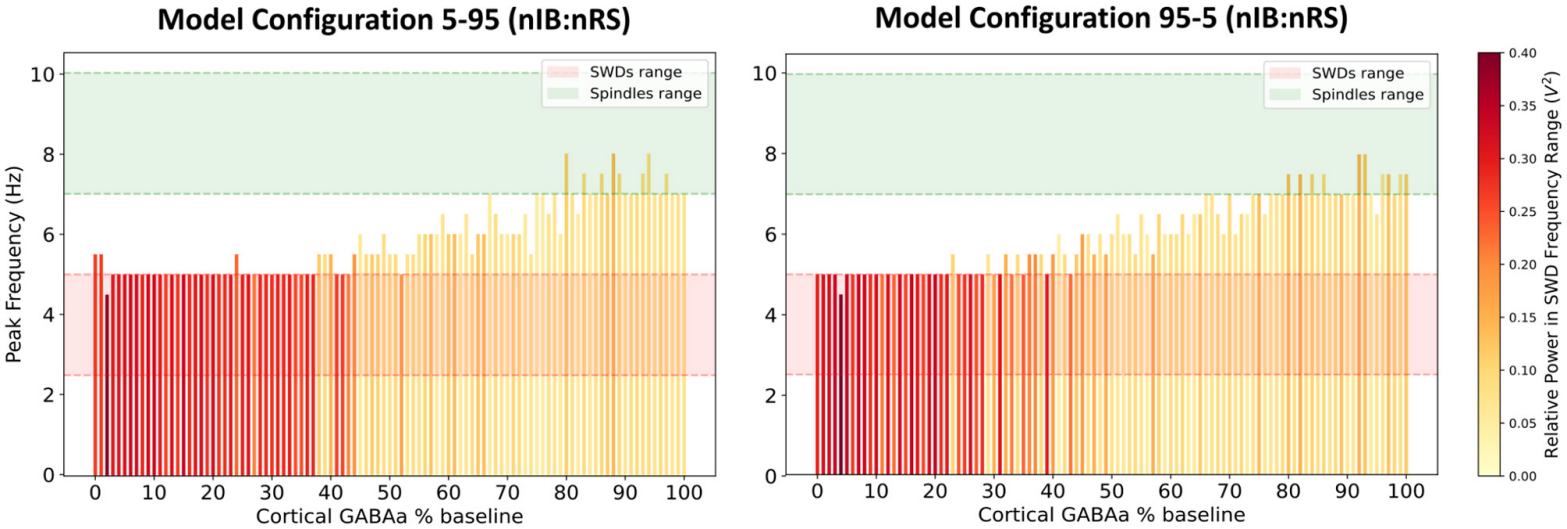
Network frequency response to varying levels of cortical GABA_A_-receptor mediated inhibition, using the 5-95 **(left)** and 95-5 **(right)** (nIB:nRS) model configurations. The color of each bar represents the relative power within the frequency range associated with SWDs.

**Figure 7:**
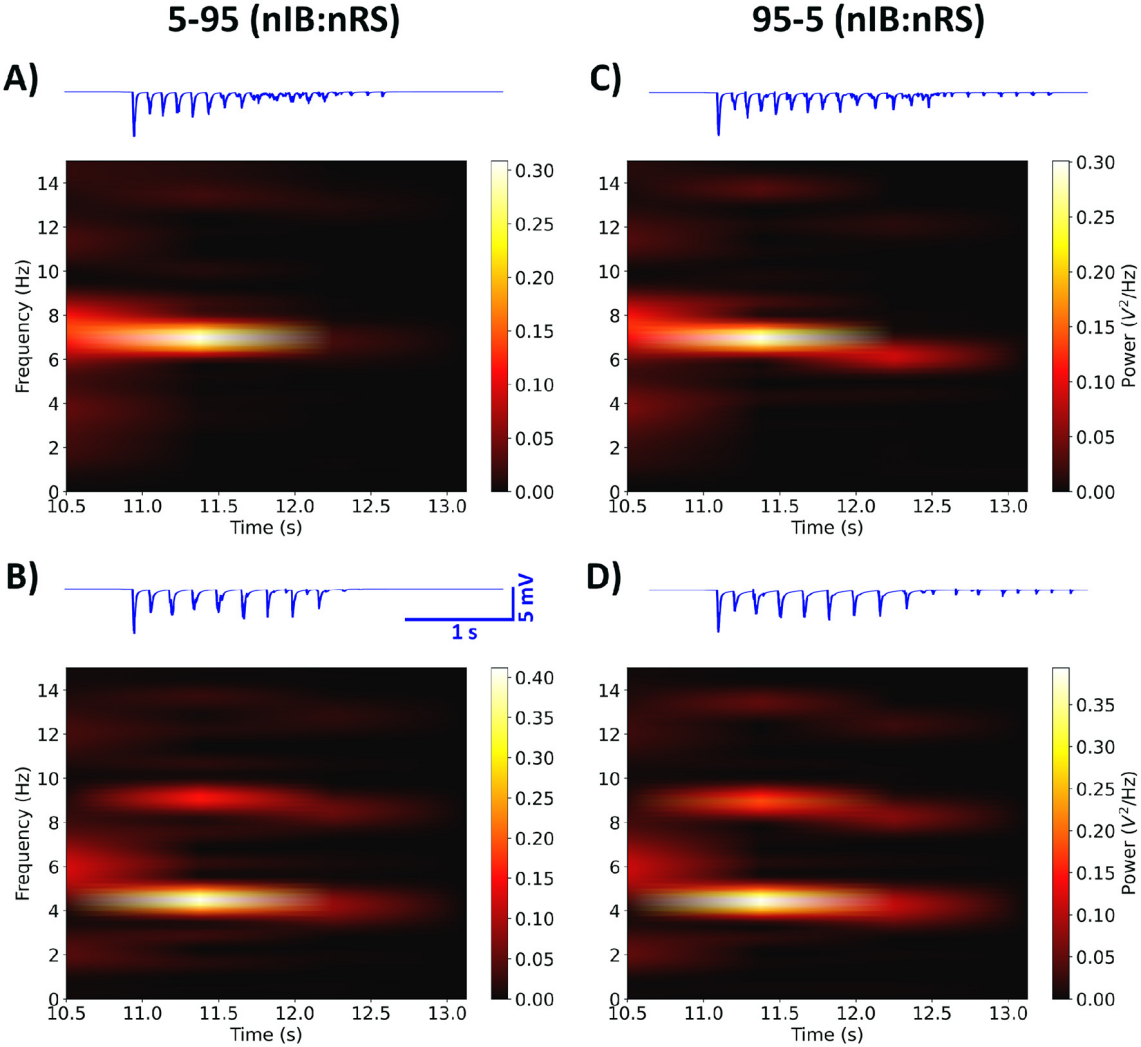
Spectral power analysis showing time-frequency representations of network activity for the 5-95 and 95-5 (nIB:nRS) model configurations, using 100% **(A, C)** and 10% **(B, D)** baseline cortical GABA_A_ conductance. For both models, fully inhibited networks exhibit maximal power at 7.5 Hz, while partially disinhibited networks show maximal power at 5 Hz. LFP traces (in blue) were calculated using the population-based method described in Section 2.2.

**Figure 8:**
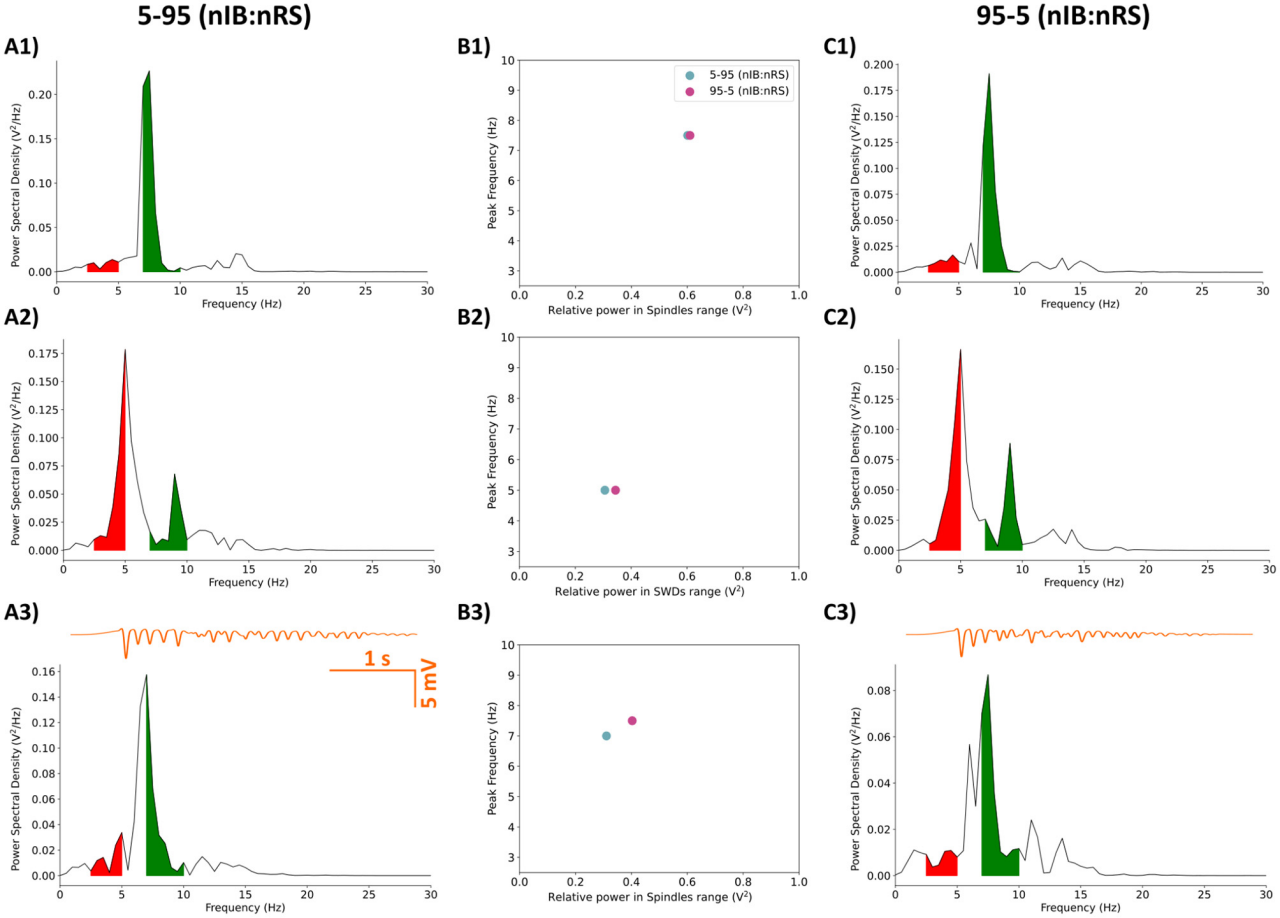
Power spectral density of the 5-95 **(A1–A3)** and 95-5 **(C1–C3)** (nIB:nRS) model configurations, calculated from filtered LFP traces (in orange). Shaded regions indicate relative power within the frequency ranges associated with SWDs (2.5–5 Hz; red) or Spindle oscillations (7–10 Hz; green). **(A1, C1)** Networks with Control synapses and full cortical inhibition, exhibiting spindle oscillations at 7.5 Hz. **(A2, C2)** Networks with Control synapses and reduced cortical GABA_A_ conductance (10% baseline), exhibiting SWDs at 5 Hz. **(A3, C3)** Networks initialized with cortical disinhibition as in **(A2, C2)** but using post-ALLO synapses, showing transition to a physiological state under the effect of ALLO. **(B1)** Relationship between relative power in spindles range and peak network frequency for fully inhibited networks. **(B2)** Relationship between relative power in SWDs range and peak network frequency for partially disinhibited networks. **(B3)** Relationship between relative power in spindles range and peak network frequency for networks under the effect of ALLO.

We first investigated the excitability profiles of both network configurations by examining network frequency responses to varying levels of GABA_A_-receptor mediated inhibition in the cortex, as shown in Figure 6. Both models display a transition from SWD to spindle activity as cortical inhibition increases. However, the thresholds at which this transition occurs differs slightly between the two, with the 95-5 (nIB:nRS) model exhibiting more intermediate oscillatory states compared to the 5-95 (nIB:nRS) model. The latter surprisingly maintains SWD activity across a broader inhibition range and requires stronger inhibition to shift to spindle-generating states. To further explore the influence of cortical composition, we simulated both networks under the same conditions as in Section 3.1—100% and 10% baseline cortical GABA_A_ conductance—to compare their abilities to sustain either physiological spindles or pathological SWDs, and to determine whether baseline dynamics differ across the two models. As shown in Figure 7, both network configurations exhibit qualitatively similar spectral characteristics, across the default mode and under cortical disinhibition. The spectral power distributions show similar frequency bands of activity (both exhibiting peak network frequencies of 7.5 Hz and 5 Hz under each condition), with only subtle differences in power distribution, likely due to variations in oscillatory patterns, as reflected in the LFP traces. This is also characterized by prominent power spectral density peaks within the 7–10 Hz (Figures 8A1, C1) and 2.5–5 Hz ranges (Figures 8A2, C2). At 100% baseline cortical GABA_A_ conductance, both models exhibit 7.5 Hz peak network frequencies with similar relative power in the spindles frequency range (Figure 8B1). Similarly, at 10% baseline conductance, both models exhibit network frequencies peaking at 5 Hz with similar relative power in the SWDs frequency range (Figure 8B2). These results suggest that despite substantial variation in cortical neuronal composition between models, the fundamental network dynamics remain conserved. This suggests that our fitted model (75-25 nIB:nRS configuration) is robust to changes in network architecture in producing baseline network states.

Following the approach in Section 2.4, we modeled the effect of ALLO in all GABA_A_ synapses, in each model configuration by first initializing to the diseased state. After implementing post-ALLO synapses in each compromised network, we observe a restorative effect, with each network transitioning back to spindle oscillations similar to the control state, as shown in Figures 8A3, C3. This transition is characterized by peak network frequencies of 7 Hz and 7.5 Hz for the 5-95 and 95-5 (nIB:nRS) models respectively, with comparable relative power in the spindles frequency range (Figure 8B3). Additionally, these qualitative restorative effects were preserved across the range of physiological and supraphysiological ALLO concentrations tested, with all levels showing ALLO-mediated resolution of SWDs, as shown in Supplementary Figure S3.

While the differences between the two model configurations is small, there is a more obvious difference between the control network states with cortical GABA_A_ at 100% and network states under the effect of ALLO. Specifically, the relative power in the spindles frequency range is higher in the control condition as compared to the post-ALLO state (Figures 8B1, B3). This is mainly due to the more sharply defined spectral density peak curves in the control condition, resulting in greater area under the curve within the spindles frequency range. In the post-ALLO state, the spectral profiles appear more spread out, resulting in relatively less concentrated power contribution falling within the spindles frequency range, despite restoring the dominant network frequency. Overall, these results suggest that, despite differences in the composition of cortical cell types (with one network having a higher proportion of IB cells), both networks respond similarly to cortical GABA_A_ modulation and the effect of ALLO, with neither showing significantly greater vulnerability to the non-resolution of SWDs.

### 3.3 Enhanced frontocortical connectivity alters SWD resolution based on network architecture

In this part of the study, we focused on modeling the connectivity differences observed in treatment non-responders prior to treatment in newly diagnosed CAE patients, particularly the increased connectivity in the frontal cortex. By simulating a 50-50 (nIB:nRS) model configuration, we aimed to investigate how, in a network with a balanced composition of cortical cell types in layer 5, increased frontocortical connectivity might influence the modulation of SWDs—particularly in the non-resolution of SWDs (i.e., a lack of effect from ALLO on SWDs). Specifically, we modeled enhanced frontal cortical connectivity by modifying the following synaptic conductance parameters with a multiplicative factor to increase synaptic strength: (i) ḡ_*IBIB*_·*F*_*IBIB*_; (ii) ḡ_*IBNRS*_·*F*_*IBNRS*_; (iii) ḡ_*NRSIB*_·*F*_*NRSIB*_. The factors used and the rationale for their selection are detailed in Section 2.5. Additionally, we explored how these enhanced synapses affect the other two model configurations [5-95 and 95-5 (nIB:nRS)] to assess whether, in addition to connectivity differences, the composition of cell types within the network influences its responsiveness to the effects of ALLO.

**Figure 9:**
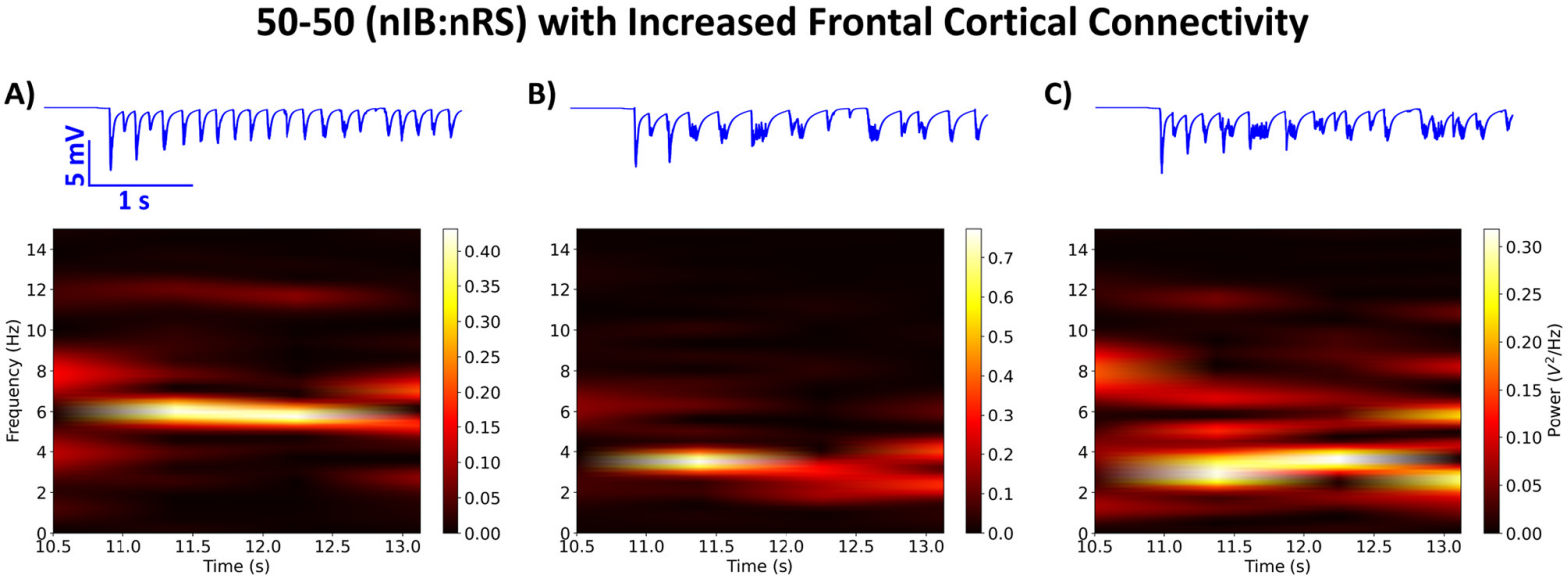
Spectral power analysis showing time-frequency representations of network activity for the 50-50 (nIB:nRS) model configuration with enhanced frontal cortical connectivity, using (*F*_*IBIB*_, *F*_*IBNRS*_, *F*_*NRSIB*_) = (7, 5, 3). **(A)** Network behavior using Control synapses and full cortical inhibition exhibits maximal power at 5.5 Hz. **(B)** Network behavior using Control synapses under partial disinhibition (10% baseline cortical GABA_A_ conductance) shows maximal power at 4 Hz. **(C)** Network initialized with cortical disinhibition as in **(B)** but using post-ALLO synapses shows a lack of restorative effect from ALLO, with a maximal frequency of 2.5 Hz. LFP traces (in blue) were calculated using the method described in Section 2.2.

Building on the approach in Section 3.1, we simulated the 50-50 (nIB:nRS) model with enhanced synaptic strengths, using (*F*_*IBIB*_, *F*_*IBNRS*_, *F*_*NRSIB*_) = (7, 5, 3). We performed the simulation under the two conditions of cortical inhibition (100% and 10% baseline conductance) to investigate the impact of these changes on network activity. As shown in Figure 9, we observed distinct changes in spectral power across different frequencies, depending on the level of cortical inhibition and the presence of ALLO. Specifically, under full cortical inhibition, the network exhibits a maximal power at 5.5 Hz (Figure 9A), marking the first instance where the peak network frequency did not coincide with the frequency exhibiting the highest power. However, the relative power within the spindles range (0.282 V^2^) was greater than that within the range associated with SWDs (0.195 V^2^). When partial disinhibition was applied, the network shifted its maximal power to 4 Hz (Figure 9B). After introducing post-ALLO synapses, the network showed a lack of a restorative effect from ALLO, with the maximal frequency reducing to 2.5 Hz (Figure 9C). The continued SWD-like behavior is further reflected in the LFP traces of network activity post-ALLO application, especially when compared to network activity under cortical disinhibition, as shown in Figures 9B, C (in blue).

**Figure 10:**
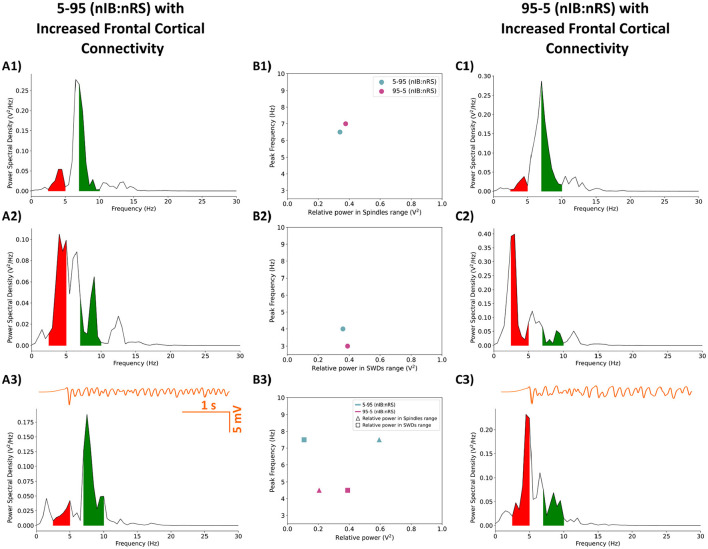
Power spectral density of the 5-95 **(A1–A3)** and 95-5 **(C1–C3)** (nIB:nRS) model configurations with enhanced frontal cortical connectivity, calculated from filtered LFP traces (in orange). Shaded regions represent relative power within frequency ranges associated with SWDs (2.5–5 Hz; red) or Spindle oscillations (7–10 Hz; green). **(A1, C1)** Networks with Control synapses and full cortical inhibition, exhibiting spindle oscillations at 6.5 Hz and 7 Hz for the 5-95 and 95-5 models, respectively. **(A2, C2)** Networks with Control synapses and reduced cortical GABA_A_ conductance (10% baseline), exhibiting SWDs at 4 Hz and 3 Hz for the 5-95 and 95-5 model, respectively. **(A3, C3)** Networks initialized with cortical disinhibition as in **(A2, C2)** but using post-ALLO synapses. The transition to a physiological state under the effect of ALLO occurs for the 5-95 model configuration, but not the 95-5 model. **(B1)** Relationship between relative power in spindles range and peak network frequency for fully inhibited networks **(B1)** and networks under the effect of ALLO **(B3)**. **(B2)** Relationship between relative power in SWDs range and peak network frequency for partially disinhibited networks.

**Figure 11:**
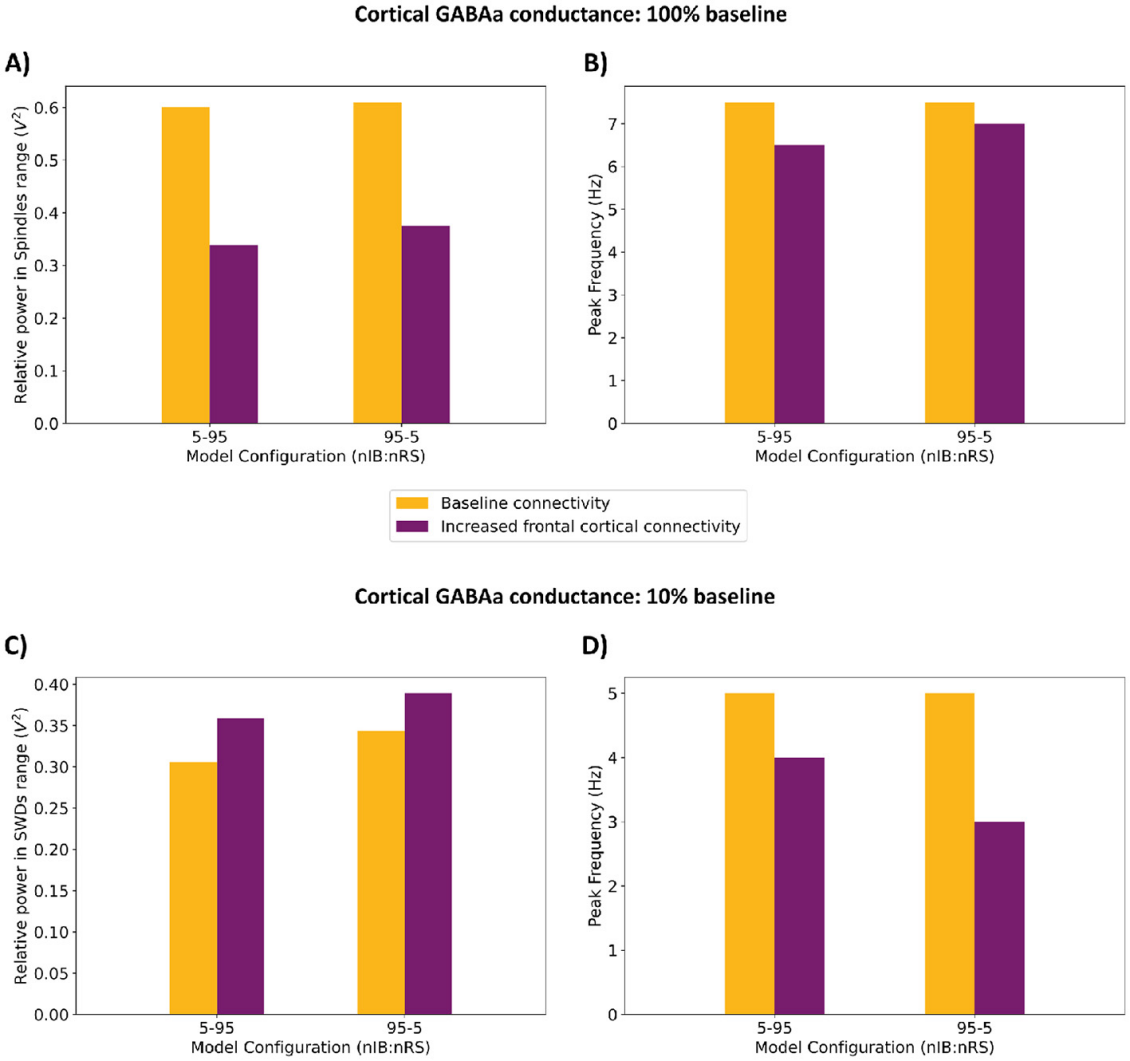
Comparison of network responses between the 5-95 and 95-5 (nIB:nRS) model configurations under two cortical GABA_A_ conductance conditions (100% and 10% baseline). **(A, C)** Relative power in the spindle/SWDs frequency range. **(B, D)** Corresponding peak frequencies of network activity. Each condition is shown under baseline (orange) and increased frontocortical connectivity (purple).

Next, we examined the impact of enhanced synaptic activity, with (*F*_*IBIB*_, *F*_*IBNRS*_, *F*_*NRSIB*_) = (7, 5, 3), on the other two model configurations, 5-95 and 95-5 (nIB:nRS). As illustrated in Figures 10A1, B1, C1, both models showed similar network frequencies [6.5 Hz for 5-95 and 7 Hz for 95-5 (nIB:nRS)] and spindle-range relative power (0.339 V^2^ and 0.376 V^2^, respectively) under the condition of 100% baseline cortical GABA_A_ conductance. Reducing cortical GABA_A_ conductance to 10% resulted in comparable network behavior across models, with peak power at 4 Hz and 3 Hz, respectively, as shown in Figures 10A2, B2, C2. Both of these findings align with the results in Section 3.2, particularly the shift from spindle oscillations to SWDs under reduced cortical inhibition. Notably, the 95-5 (nIB:nRS) model consistently exhibited higher relative power across both frequency ranges, regardless of frontocortical connectivity alterations, as illustrated in Figures 11A, C. Without enhanced synaptic activity, network frequencies were identical between models and changed similarly across levels of cortical inhibition, as shown in Figures 11B, D (orange bars). However, when frontocortical connectivity was enhanced, the 95-5 (nIB:nRS) model demonstrated higher frequency under full inhibition and a lower frequency under partial disinhibition, as illustrated in Figures 11B, D (purple bars).

Introducing post-ALLO synapses in networks initialized to a pathological state, however, leads to interesting differences between the models. Surprisingly, the effect of ALLO on the 5-95 model mirrors the results from Section 3.2, where ALLO exerts a restorative effect, shifting the network's maximal power into the spindles range with a peak network frequency of 7.5 Hz, as shown in Figure 10A3. This shift occurs despite there being enhanced frontal cortical connectivity. In contrast, the effect of ALLO on the 95-5 (nIB:nRS) model resembles that seen in the 50-50 (nIB:nRS) model, with maximal power in the SWD-associated frequency range and a peak network frequency of 4.5 Hz.

## 4 Discussion

In this study, we developed a simplified thalamocortical model with a layered cortical structure to investigate how variations in frontocortical connectivity might influence the effectiveness of ALLO in resolving SWDs in CAE. We modeled the cortical regions with specific neuron firing patterns, using IB neurons to represent the frontal cortex and RS neurons to represent the parietal cortex. By exploring two cortical compositions, 5-95 (nIB:nRS) and 95-5 (nIB:nRS), we examined circuits with differing contributions from the frontal and parietal regions. This model allowed us to investigate how neurosteroid modulation, specifically ALLO, interacts with varying cortical compositions to influence seizure dynamics and treatment outcomes.

Our results demonstrate that both physiological and pathological oscillations are maintained by the thalamocortical circuit regardless of which model configuration is used [5-95 or 95-5 (nIB:nRS) model]. Under different conditions of cortical inhibition, both models exhibited similar network behavior, promoting spindles and SWD activity. This finding aligns with the classification of absence seizures as a generalized epilepsy type, where activity that may originate in one region, such as the parietal cortex, can recruit other cortical areas to sustain seizure activity (Meeren et al., 2002; Polack et al., 2007). Similarly, sleep spindles are known to vary in frequency and cortical source across different brain regions (Purcell et al., 2017). The most striking finding from our study came when we introduced ALLO under conditions of increased frontocortical connectivity. ALLO, a neurosteroid that potentiates GABA_A_-receptor function, gradually increases during puberty (Fadalti et al., 1999), a period that coincides with the remission of CAE in many cases (van Luijtelaar et al., 2001). In our simulations, both models showed a restorative effect of ALLO, helping them recover from the SWD state at baseline connectivity. However, only the 5-95 (nIB:nRS) model was able to recover when frontocortical connectivity was enhanced. This differential response to ALLO is particularly notable, considering that both models initially display similar behavior under physiological and pathological states. This suggests that individuals with different neuronal composition profiles may follow distinct clinical trajectories despite similar initial presentations. Specifically, our results suggest that patients with connectivity profiles resembling the 5-95 network (i.e., parietal-dominant configuration) may experience remission following hormonal changes involving ALLO, whereas those with profiles resembling the 95-5 network (i.e., frontal-dominant configuration) may be predisposed to non-remission.

Our results suggest that non-resolving CAE may result not only from increased strength of connections in the frontal cortex but also from the composition of cell types within the network, with a higher proportion of bursting-type cells preventing the therapeutic effects of ALLO. This highlights the significance of individual neuronal components in network dynamics—much like the concept of ictogenic neurons, where even in a network with largely non-pathological connectivity, specific neuronal populations can contribute to pathological activity patterns (Depaulis and Charpier, 2018; Polack et al., 2007). Our findings are consistent with clinical studies using functional connectivity analysis that have identified pre-treatment ictal connectivity differences between patients who ultimately experience remission and those who do not. In particular, increased frontal cortical connectivity has been observed in patients with non-remitting seizures, and treatment non-responders (Tenney et al., 2018). It is important to note that these clinical studies employ functional connectivity analyses, whereas our modeling involves structural connectivity alterations. Functional connectivity measures the relationship between neural activity in different brain regions, without directly reflecting physical connections (Straathof et al., 2019). On the contrary, structural connectivity in our model refers to the actual synaptic connections between neurons. Despite this difference in approach, our ability to reproduce similar results by modifying synaptic strengths suggests that structural alterations may underlie the functional connectivity patterns observed clinically. This provides a potential mechanistic explanation for the clinical heterogeneity observed in CAE outcomes.

Some of the limitations of this work include our simplified representation of frontal and parietal cortices, with neuronal firing types (bursting vs. regular spiking) serving as the primary distinguishing feature between cortical regions. In reality, both firing types exist across cortical regions, albeit in different proportions (Chagnac-Amitai et al., 1990; Yang et al., 1996). Future models could be improved by incorporating additional distinguishing features of these regions, such as region-specific distributions of receptors mediating excitation or inhibition. In addition, future extensions of the model could incorporate basal ganglia-thalamocortical loop interactions, including thalamic-subthalamic and striatal-cortical projections, which have been shown to influence the resolution of SWDs (Hu et al., 2024, 2025). It is also important to note that the role of ALLO in modulating basal ganglia function has been demonstrated in several other neurological conditions, where ALLO's actions are predominantly beneficial (Nezhadi et al., 2016; Irwin et al., 2015). These effects reflect ALLO's ability to enhance GABAergic inhibition, exert neuroprotective actions, and stabilize network activity within basal ganglia-thalamocortical circuits. Building on these mechanisms, one could further examine whether increased frontocortical connectivity as a potential mechanism of resistance to ALLO-mediated resolution of SWDs also applies to CAE models that incorporate subthalamic or broader basal ganglia structures, or whether such model extensions instead provide a more robust compensatory pathway capable of sustaining SWD resolution.

Furthermore, CAE pathophysiology is known to involve a range of genetic mechanisms beyond the reduced cortical inhibition linked to *GABRG*2 mutations, which may interact differently with ALLO and connectivity profiles. For example, in our previous work using a conductance-based thalamocortical model, we found that networks initialized with altered T-type *Ca*^2+^ conductance, suggestive of mutations in the *CACNA* gene, showed reduced efficacy of ALLO in resolving SWDs (Ahmed and Campbell, 2024). While this highlights one potential mechanism for non-resolution, the focus of this study is to explore whether certain genetic mechanisms may better compensate for increased frontocortical connectivity to facilitate the resolution of SWD activity. To account for the multigenetic nature of CAE, ongoing work includes investigating whether connectivity differences that prevent SWD resolution are consistent across other genetic mechanisms, particularly variants of the *SCN* gene.

Our model provides a solid foundation that can be adapted in future studies to explore chronic ALLO exposure, long-term network plasticity, and developmental changes in connectivity—processes that are critical for sustained remission in adolescence. Given that the model is positioned in a drug-naive setting, it is well-suited for investigating differential drug responses based on connectivity profiles. Future studies could test whether specific pharmacological interventions are better suited for particular connectivity patterns, inferred through the analysis of non-invasive neuroimaging data, such as fMRI or EEG. This would extend existing computational studies that evaluate treatment responses in models with specific genetic mechanisms underlying the disease state, providing insights into potential treatment response trajectories (Knox et al., 2018). In addition, use of PET/MRI imaging with specific PET tracers could enable visualization of the expression of different GABA receptor subtypes and the investigation of pre-treatment correlations between sex steroids and the GABA neurotransmitter system. Furthermore, in collaboration with experimentalists, patient-derived induced pluripotent stem cell (iPSC)-based neuronal cultures could be used to validate key model predictions, such as the responsiveness of ALLO to different neuronal compositions within the cultures. These efforts would be crucial for understanding how approaches such as chemogenetics and optogenetics can be used to modulate neuronal circuits in a cell-type and region-specific manner, in combination with targeted delivery or modulation of steroid hormones, to develop personalized therapeutic strategies.

## Data Availability

The raw data supporting the conclusions of this article will be made available by the authors, without undue reservation.
