# Supplementary material for: Computational modeling of resistance to hormone-mediated remission in childhood absence epilepsy
Source: Front Comput Neurosci. 2026 Jan 12;19:1733650. doi: 10.3389/fncom.2025.1733650 (PMC12833357; doi:10.3389/fncom.2025.1733650)
Supplement: Supplementary file 1 [file Data_Sheet_1.pdf]

Supplementary Material

1 MODEL COMPONENTS

All of the neurons were described using a single compartment representing the soma. The soma was modelled as a cylinder with a surface area of πdl, where d is the diameter and l is the length. The diameter was 18 μm for both IB and RS neurons, 16 μm for NRS and LTS neurons, 96 μm for TC neurons, and 70 μm for all RE neurons. The length was 25 μm for both IB and RS cells, 20 μm for NRS and LTS cells, 96 μm for TC cells, and 64.86 μm for RE neurons. The axial resistance, Ra was equal to 250 Ωcm for all cortical cells and 100 Ωcm for both thalamic neurons. The membrane conductance density distributions as well as the type of current differed between neuron type. Ionic currents were described with the general form given by the following equation:

$$I_{ion} = \bar{g}_{ion} \cdot m^N \cdot h^M \cdot (V - V_{eq}) \tag{S1}$$

where $\bar{g}_{ion}$ represents the maximal conductance for a particular ion channel, m and h are gating variables, N and M are integer powers, and V_{eq} represents the equilibrium potential. The dynamics of the gating variables were described by one of two general forms: rise and decay rate functions, or a Boltzmann function combined with a voltage-dependent time constant function.

Using rise and decay rate functions, the dynamics were described by the following form:

$$\frac{dx}{dt} = \frac{x_\infty - x}{\tau_x} \tag{S2}$$

$$x_\infty = \frac{\alpha_x}{\alpha_x + \beta_x} \quad \text{and} \quad \tau_x = \frac{1}{\alpha_x + \beta_x} \tag{S3}$$

where x_∞ is the steady-state gating variable function, τ_x represents the time course for approaching the steady state, α_x and β_x represent the forward and backward rate functions.

Alternatively, the gating variable dynamics were described by the following Boltzmann function:

$$x_\infty = \frac{1}{1 + \exp(\frac{V_{1/2} - V}{k})} \tag{S4}$$

where x_∞ is the steady-state gating variable function, V is the membrane potential, $V_{1/2}$ is the voltage at half-activation, and k is the slope factor representing the steepness of the voltage-dependent transition between closed and open states of the channel. The voltage-dependent time constant function (τ_x) describes how quickly the gating variable approaches steady state and its form varies according to the channel type.

The incorporated Na^+, K^+, and Ca^{2+} currents included in the model are summarized in Supplementary Table S1.

The equilibrium potentials were defined as follows: $V_{Na} = 50$ mV (all cells), $V_K = -100$ mV (for LTS, TC, RE) and -95 mV (for RS, IB, NRS), $V_h = -40$ mV (for LTS, TC, RE) and -43 mV (for RS, IB, NRS), $V_{Leak} = -70$ mV (for RS, IB, NRS, TC), -65 mV for LTS and -90 mV for RE neurons, V_{Ca} was given by intracellular calcium dynamics described in Supplementary Section 1.2.1.

Table S1. Description of the currents used for all neuron types.

Current	Current Description
I_{Naf}	fast and transient inactivating Na^+ current
I_{Nap}	persistent Na^+ current
I_K	fast K^+ current
I_{Kdr}	delayed rectifier K^+ current
I_{Ka}	transient inactivating K^+ current
I_{K2}	slowly activating and inactivating K^+ current
I_{Km}	muscarinic receptor-suppressed K^+ current
I_{Kc}	fast voltage and Ca^{2+}-dependent K^+ current
I_{Kahp}	slow Ca^{2+}-dependent K^+ current
I_{CaT}	low-threshold Ca^{2+} current
I_{CaL}	high-threshold Ca^{2+} current
I_h	slowly non-inactivated mixed Na^+/K^+ current
I_{Leak}	leak current
I_{KLeak}	leak K^+ current

1.1 Summary of currents

1.1.1 I_{Leak}

The leak current for all neuron types was described as a simple ohmic current:

$$I_{Leak} = g_{Leak} \cdot (V - V_{Leak}) \tag{S5}$$

where g_{Leak} is the constant conductance.

1.1.2 I_{KLeak}

The leak K^+ current in TC neurons was described by the following Ohmic equation:

$$I_{KLeak} = g_{KLeak} \cdot (V - V_K) \tag{S6}$$

where g_{KLeak} is the maximal conductance.

1.1.3 I_{Naf}

The fast Na^+ current was described using a Hodgkin-Huxley style equation:

$$I_{Naf} = g_{Naf} \cdot m_{Naf}^3 \cdot h_{Naf} \cdot (V - V_{Na}) \tag{S7}$$

where g_{Naf} is the maximal conductance, m_{Naf} is the activation variable, and h_{Naf} is the inactivation variable.

In neurons of the RS, IB and NRS type, kinetics were based on dynamics described in Traub et al. (Traub et al., 2005):

$$m_{Naf\infty} = \frac{1}{1 + \exp\left(\frac{-V-38}{10}\right)} \tag{S8}$$

$$\tau_{m_{Naf}} = \begin{cases} 0.025 + 0.14 \cdot \exp\left(\frac{V+30}{10}\right), & V \leq -30\text{mV} \\ 0.02 + 0.145 \cdot \exp\left(\frac{-V-30}{10}\right), & V > -30\text{mV} \end{cases} \tag{S9}$$

$$h_{Naf\infty} = \frac{1}{1 + \exp\left(\frac{V+62.9}{10.7}\right)} \tag{S10}$$

$$\tau_{h_{Naf}} = 0.15 + \frac{1.15}{1 + \exp\left(\frac{V+37}{15}\right)} \tag{S11}$$

In LTS neurons, the steady state activation function, $m_{Naf\infty}$, was the same as described above for other cortical neurons, while the remaining kinetics were based on dynamics described in Martina and Jonas (Martina and Jonas, 1997):

$$\tau_{m_{Naf}} = \begin{cases} 0.0125 + 0.1525 \cdot \exp\left(\frac{V+30}{10}\right), & V \leq -30\text{mV} \\ 0.02 + 0.145 \cdot \exp\left(\frac{-V-30}{10}\right), & V > -30\text{mV} \end{cases} \tag{S12}$$

$$h_{Naf\infty} = \frac{1}{1 + \exp\left(\frac{V+58.3}{6.7}\right)} \tag{S13}$$

$$\tau_{h_{Naf}} = 0.225 + \frac{1.125}{1 + \exp\left(\frac{V+37}{15}\right)} \tag{S14}$$

In TC and RE neurons, the kinetics for this current were based on dynamics described in Traub and Miles (Traub et al., 1991):

$$\alpha_{m_{Naf}} = \frac{0.32 \cdot (-V - 50)}{\exp\left(\frac{-V-50}{4}\right) - 1} \tag{S15}$$

$$\beta_{m_{Naf}} = \frac{0.28 \cdot (V + 23)}{\exp\left(\frac{V+23}{5}\right) - 1} \tag{S16}$$

$$\alpha_{h_{Naf}} = 0.128 \cdot \exp\left(\frac{-V - 46}{18}\right) \tag{S17}$$

$$\beta_{h_{Naf}} = \frac{4}{1 + \exp\left(\frac{-V-23}{5}\right)} \tag{S18}$$

1.1.4 I_{Nap}

The persistent, depolarization-activated Na^+ current was described using kinetics presented by Traub et al. (Traub et al., 2003):

$$I_{Nap} = g_{Nap} \cdot m_{Nap} \cdot (V - V_{Na}) \tag{S19}$$

where g_{Nap} is the maximal conductance, and m_{Nap} is the activation variable. For all cortical neurons, the steady state activation function and decay time constant were described by the following:

$$m_{Nap\infty} = \frac{1}{1 + \exp\left(\frac{-V-48}{10}\right)} \tag{S20}$$

$$\tau_{m_{Nap}} = \begin{cases} 0.025 + 0.14 \cdot \exp\left(\frac{V+40}{10}\right), & V \leq -40\text{mV} \\ 0.02 + 0.145 \cdot \exp\left(\frac{-V-40}{10}\right), & V > -40\text{mV} \end{cases} \tag{S21}$$

1.1.5 I_K

The fast K^+ current in both thalamic cells was described using the following equation:

$$I_K = g_K \cdot n_K^4 \cdot (V - V_K) \tag{S22}$$

where g_K is the maximal conductance, n_K is the inactivation variable. All functions were based on dynamics described in Traub et al. (Traub et al., 1991):

$$\alpha_{n_K} = \frac{0.032 \cdot (-V - 48)}{\exp(\frac{-V-48}{5}) - 1} \tag{S23}$$

$$\beta_{n_K} = 0.5 \cdot \exp\left(\frac{-V - 53}{40}\right) \tag{S24}$$

1.1.6 I_{Kdr}

The delayed rectifier K^+ current was described using kinetics presented by Traub et al. (Traub et al., 2003):

$$I_{Kdr} = g_{Kdr} \cdot m_{Kdr}^4 \cdot (V - V_K) \tag{S25}$$

where g_{Kdr} is the maximal conductance and m_{Kdr} is the activation variable.

In neurons of the RS, IB and NRS type, the steady state activation function was described by the following:

$$m_{Kdr\infty} = \frac{1}{1 + \exp\left(\frac{-V-29.5}{10}\right)} \tag{S26}$$

In LTS neurons, the steady activation function was defined as:

$$m_{Kdr\infty} = \frac{1}{1 + \exp\left(\frac{-V-27}{11.5}\right)} \tag{S27}$$

The time constant for all cortical neurons was described by the following:

$$\tau_{m_{Kdr}} = \begin{cases} 0.25 + 4.35 \cdot \exp\left(\frac{V+10}{10}\right), & V \leq -10\text{mV} \\ 0.25 + 4.35 \cdot \exp\left(\frac{-V-10}{10}\right), & V > -10\text{mV} \end{cases} \tag{S28}$$

1.1.7 I_{Ka}

The transient inactivating K^+ current was modelled in all cortical cells using the following equation:

$$I_{Ka} = g_{Ka} \cdot m_{Ka}^4 \cdot h_{Ka} \cdot (V - V_K) \tag{S29}$$

where g_{Ka} is the maximal conductance, and m_{Ka} and h_{Ka} are the activation and inactivation variables, respectively. The kinetics were based on dynamics described by Huguenard and McCormick (Huguenard and McCormick, 1992):

$$m_{Ka\infty} = \frac{1}{1 + \exp\left(\frac{-V-60}{8.5}\right)} \tag{S30}$$

$$\tau_{m_{Ka}} = 0.185 + \frac{0.5}{\exp\left(\frac{V+35.8}{19.7}\right) + \exp\left(\frac{-V-79.7}{12.7}\right)} \tag{S31}$$

$$h_{Ka\infty} = \frac{1}{1 + \exp\left(\frac{V+78}{6}\right)} \tag{S32}$$

$$\tau_{m_{Naf}} = \begin{cases} \frac{0.5}{\exp\left(\frac{V+46}{5}\right) + \exp\left(\frac{-V-238}{37.5}\right)}, & V \leq -63\text{mV} \\ 9.5, & V > -63\text{mV} \end{cases} \tag{S33}$$

1.1.8 I_{K2}

The slowly activating and inactivating K^+ current equations followed Huguenard and McCormick (Huguenard and McCormick, 1992) and McCormick and Huguenard (McCormick and Huguenard, 1992):

$$I_{K2} = g_{K2} \cdot m_{K2} \cdot h_{K2} \cdot (V - V_K) \tag{S34}$$

where g_{K2} is the maximal conductance, and m_{K2} and h_{K2} are the activation and inactivation variables, respectively. The kinetics for all cortical neurons were described by the following equations:

$$m_{K2\infty} = \frac{1}{1 + \exp\left(\frac{-V-10}{17}\right)} \tag{S35}$$

$$\tau_{m_{K2}} = 4.95 + \frac{0.5}{\exp\left(\frac{V-81}{25.6}\right) + \exp\left(\frac{-V-132}{18}\right)} \tag{S36}$$

$$h_{K2\infty} = \frac{1}{1 + \exp\left(\frac{V+58}{10.6}\right)} \tag{S37}$$

$$\tau_{h_{K2}} = 60 + \frac{0.5}{\exp\left(\frac{V-1.33}{200}\right) + \exp\left(\frac{-V-130}{7.1}\right)} \tag{S38}$$

(S39)

1.1.9 I_{Km}

The non-inactivating, slow voltage-dependent K^+ current was modelled using the following equation:

$$I_{Km} = g_{Km} \cdot m_{Km} \cdot (V - V_K) \tag{S40}$$

where g_{Km} is the maximal conductance, and m_{Km} is the activation variable.

In all cortical neurons, kinetics were based on dynamics presented by Traub et al. (Traub et al., 2003):

$$\alpha_{m_{Km}} = \frac{0.02}{1 + \exp\left(\frac{-V-20}{5}\right)} \tag{S41}$$

$$\beta_{m_{Km}} = 0.5 \cdot \exp\left(\frac{-V-43}{18}\right) \tag{S42}$$

In TC and RE neurons, the kinetics were based on dynamics described in McCormick et al. (McCormick et al., 1993):

$$m_{Km_\infty} = \frac{1}{1 + \exp\left(\frac{-V-35}{10}\right)} \tag{S43}$$

$$\tau_{m_{Km}} = \frac{1000}{3.3 \cdot \left(\exp\left(\frac{V+35}{20}\right) + \exp\left(\frac{-V-35}{20}\right)\right)} \tag{S44}$$

1.1.10 I_{Kc}

The fast voltage and Ca^{2+}-dependent K^+ current was modelled using the equation presented by Traub et al. (Traub et al., 1994):

$$I_{Kc} = g_{Kc} \cdot m_{Kc} \cdot \min(0.004[Ca^{2+}]_\mathrm{i}, 1) \cdot (V - V_K) \tag{S45}$$

where g_{Kc} is the maximal conductance, m_{Kc} is the activation variable, and $[Ca^{2+}]_\mathrm{i}$ dynamics are governed by dynamics described in 1.2.

The forward and backward rate functions for all neurons of the RS, IB, and NRS type were based on kinetics described in Traub et al. (Traub et al., 2003):

$$\alpha_{m_{Kc}} = \begin{cases} 0.053 \cdot \exp\left(\frac{V+50}{11} - \frac{V+53.5}{27}\right), & V \leq -10\text{mV} \\ 2 \cdot \exp\left(\frac{-V-53.5}{27}\right), & V > -10\text{mV} \end{cases} \tag{S46}$$

$$\beta_{m_{Kc}} = \begin{cases} 2 \cdot \exp\left(\frac{-V-53.5}{27}\right) - \alpha_{m_{Kc}}, & V \leq -10\text{mV} \\ 0, & V > -10\text{mV} \end{cases} \tag{S47}$$

For LTS neurons, the forward and backward rate functions as described above were multiplied by a factor of 2.

1.1.11 I_{Kahp}

The slow Ca^{2+}-dependent K^+ current responsible for afterhypolarization, was described using kinetics presented by Traub et al. (Traub et al., 1994):

$$I_{Kahp} = g_{Kahp} \cdot m_{Kahp} \cdot (V - V_K) \tag{S48}$$

where g_{Kahp} is the maximal conductance, and m_{Kahp} is the activation variable. The forward rate function for all cortical neurons was dependent on the intracellular calcium dynamics described in Supplementary

Section 1.2:

$$\alpha_{m_{Kahp}} = \min(0.0001[Ca^{2+}]_{\mathrm{i}}, 0.01) \tag{S49}$$

$$\beta_{m_{Kahp}} = 0.01 \tag{S50}$$

1.1.12 I_{CaT}

The low-threshold Ca^{2+} current was modelled using the following equation:

$$I_{CaT} = g_{CaT} \cdot m_{CaT}^2 \cdot h_{CaT} \cdot (V - V_{Ca}) \tag{S51}$$

where g_{CaT} is the maximal conductance, m_{CaT} and h_{CaT} are the activation and inactivation gating variables, respectively. The dynamics were described differently for each thalamic neuron, as well as within the cortical cells.

For cortical neurons of the RS, IB, and NRS type, the kinetics were based on descriptions by Traub et al. (Traub et al., 2003):

$$m_{CaT\infty} = \frac{1}{1 + \exp\left(\frac{-V-56}{6.2}\right)} \tag{S52}$$

$$\tau_{m_{CaT}} = 0.204 + \frac{0.333}{\exp\left(\frac{-V-131}{16.7}\right) + \exp\left(\frac{V+15.8}{18.2}\right)} \tag{S53}$$

$$h_{CaT\infty} = \frac{1}{1 + \exp\left(\frac{80}{4}\right)} \tag{S54}$$

$$\tau_{h_{CaT}} = \begin{cases} 0.333 \cdot \exp\left(\frac{V+466}{66.6}\right), & V \leq -81\mathrm{mV} \\ 9.32 + 0.333 \cdot \exp\left(\frac{-V-21}{10.5}\right), & V > -81\mathrm{mV} \end{cases} \tag{S55}$$

For the LTS interneuron, kinetics were based on descriptions by Destexhe et al. (Destexhe et al., 1996b):

$$m_{CaT\infty} = \frac{1}{1 + \exp\left(\frac{-V-52}{7.4}\right)} \tag{S56}$$

$$\tau_{m_{CaT}} = 1 + \frac{0.333}{\exp\left(\frac{V+27}{10}\right) + \exp\left(\frac{-V-102}{15}\right)} \tag{S57}$$

$$h_{CaT\infty} = \frac{1}{1 + \exp\left(\frac{V+80}{5}\right)} \tag{S58}$$

$$\tau_{h_{CaT}} = 28.3 + \frac{0.33}{\exp\left(\frac{V+48}{4}\right) + \exp\left(\frac{-V-407}{50}\right)} \tag{S59}$$

For RE neurons, kinetics were slightly modified from the description for LTS cells, as given by Traub et al. (Traub et al., 2005):

$$m_{CaT\infty} = \frac{1}{1 + \exp\left(\frac{-V-50}{7.4}\right)} \tag{S60}$$

$$\tau_{m_{CaT}} = \frac{3 + \frac{1}{\exp\left(\frac{V+25}{10}\right) + \exp\left(\frac{-V-100}{15}\right)}}{2.5^{1.2}} \tag{S61}$$

$$h_{CaT\infty} = \frac{1}{1 + \exp\left(\frac{V+78}{5}\right)} \tag{S62}$$

$$\tau_{h_{CaT}} = \frac{85 + \frac{1}{\exp\left(\frac{V+48}{4}\right) + \exp\left(\frac{-V-407}{50}\right)}}{2.5^{1.2}} \tag{S63}$$

For TC neurons, activation m_{CaT} was taken to be at steady-state equal to $m_{CaT\infty}$, and all steady-state activation and inactivation functions were based on the description by Huguenard and McCormick (Huguenard and McCormick, 1992), and the inactivation time constant was as described by Destexhe et al. (Destexhe et al., 1996a):

$$m_{CaT\infty} = \frac{1}{1 + \exp\left(\frac{-V-57}{6.2}\right)} \tag{S64}$$

$$h_{CaT\infty} = \frac{1}{1 + \exp\left(\frac{V+81}{4}\right)} \tag{S65}$$

$$\tau_{h_{CaT}} = \frac{30.8 + \frac{211.4 + \exp\left(\frac{V+113.2}{5}\right)}{1 + \exp\left(\frac{V+84}{3.2}\right)}}{3^{1.2}} \tag{S66}$$

1.1.13 I_{CaL}

The high-threshold Ca^{2+} current was modelled using the following equation:

$$I_{CaL} = g_{CaL} \cdot m_{CaL}^2 \cdot (V - V_{Ca}) \tag{S67}$$

where g_{CaL} is the maximal conductance, and m_{CaL} is the activation variable. The forward and backward rate function for all cortical neurons were as described by Traub et al. (Traub et al., 2003):

$$\alpha_{m_{CaL}} = \frac{1.6}{1 + \exp\left(-0.072 \cdot (V - 5)\right)} \tag{S68}$$

$$\beta_{m_{CaL}} = 0.1 \cdot \frac{\frac{V+8.9}{5}}{\exp\left(\frac{V+8.9}{5}\right) - 1} \tag{S69}$$

1.1.14 I_h

The mixed Na^+/K^+ cation current was modelled differently for cortical cells and the TC cell. For all cortical cells, this was modelled using the following equation:

$$I_h = g_h \cdot m_h \cdot (V - V_h) \tag{S70}$$

where g_h is the maximal conductance, and m_h is the voltage-dependent activation variable, based on the description by Huguenard and McCormick (Huguenard and McCormick, 1992):

$$m_{h\infty} = \frac{1}{1 + \exp\left(\frac{V+75}{5.5}\right)} \tag{S71}$$

$$\tau_{m_h} = \frac{1}{\exp(-14.59 - 0.086 \cdot V) + \exp(-1.87 + 0.0701 \cdot V)} \tag{S72}$$

For TC cells, in addition to being voltage-dependent, the current dynamics were also dependent on intracellular Ca^{2+} concentration:

$$I_h = g_h \cdot ([O] + 2 \cdot [O_L]) \cdot (V - V_h) \tag{S73}$$

where g_h is the maximal conductance, and the activation variable, m_h, is replaced by a weighted sum of channels in the open form and calcium-bound open form, based on the kinetic scheme described by Destexhe et al. (Destexhe et al., 1996a):

$$C \underset{\beta(V)}{\overset{\alpha(V)}{\rightleftharpoons}} O \tag{S74}$$

$$P_0 + 2Ca_i^{2+} \underset{k_2}{\overset{k_1}{\rightleftharpoons}} P_1 \tag{S75}$$

$$O + P_1 \underset{k_4}{\overset{k_3}{\rightleftharpoons}} O_L \tag{S76}$$

where voltage-dependent transitions between closed (C) and open (O) forms occur with rates described by $\alpha(V)$ and $\beta(V)$, and intracellular Ca^{2+} ions bind to a regulating factor given by P_0 (unbound) and P_1 (bound) with rates of $k_1 = 2.5 \times 10^7$ mM^{-4}ms^{-1} and $k_2 = 4 \times 10^{-4}$ ms^{-1}. The dynamics of Ca^{2+} ions is described in Supplementary Section 1.2. Furthermore, the open form of the channel binds with the calcium-bound regulator P_1 to form O_L with rates $k_3 = 0.1$ ms^{-1} and $k_4 = 0.001$ ms^{-1}.

The voltage-dependent transition rates between closed and open forms are described by:

$$\alpha(V) = \frac{m_{h\infty}}{\tau_{m_h}} \tag{S77}$$

$$\beta(V) = \frac{1 - m_{h\infty}}{\tau_{m_h}} \tag{S78}$$

where

$$m_{h\infty} = \frac{1}{1 + \exp\left(\frac{V+75}{5.5}\right)} \tag{S79}$$

$$\tau_{m_h} = 20 + \frac{1000}{\exp\left(\frac{-V-89}{11.6}\right) + \exp\left(\frac{V+71.5}{14.2}\right)} \tag{S80}$$

1.2 Calcium dynamics

Intracellular calcium dynamics were based on the model described by Destexhe et al. (Destexhe et al., 1993). A simple proportional model of Ca^{2+} diffusion was used with the change in calcium concentration described by:

$$\frac{d[Ca^{2+}]_{\mathrm{i}}}{dt} = -\frac{k}{2Fd} \cdot I_T + \frac{[Ca^{2+}]_{\mathrm{i}\infty} - [Ca^{2+}]_{\mathrm{i}}}{\tau_r} \tag{S81}$$

where $[Ca^{2+}]_{\mathrm{i}}$ is measured in mM, F = 96489 Cmol^{-1} is Faraday's constant, d denotes the depth at which calcium concentration was calculated beneath the membrane, $k = 10^{-4}$ is a dimensionless unit conversion factor, I_T is the calcium current measured in mA/cm^2, $[Ca^{2+}]_{\mathrm{i}\infty} = 2.4 \times 10^{-4}$ mM is the steady state calcium concentration, and τ_r is the decay time constant. The depth at which the calcium concentration was calculated was $12 \times 10^{-3}\mu$m for the IB and RS neuron, $4 \times 10^{-3}\mu$m for the NRS neuron, $2 \times 10^{-4}\mu$m for the LTS neuron, and 1μm for both the TC and RE neurons. The decay time constants were 100ms for cortical cells of the IB, RS, and NRS type, 50 ms for the LTS type, and 5 ms for both TC and RE cells.

1.2.1 Equilibrium potential for Ca^{2+}

The equilibrium potential for calcium, V_{Ca} was calculated according to the Nernst relation described in (Destexhe et al., 1993):

$$V_{Ca} = k' \frac{RT}{2F} \log \frac{[Ca^{2+}]_{\mathrm{o}}}{[Ca^{2+}]_{\mathrm{i}}} \tag{S82}$$

where $R = 8.31$ J mol^{-1}K^{-1}, $T = 309.15$°K, $F = 96489$ Cmol^{-1} is Faraday's constant, $[Ca^{2+}]_{\mathrm{o}}$ and $[Ca^{2+}]_{\mathrm{i}}$ denote extracellular and intracellular calcium concentrations respectively, and $k' = 1000$ is a unit conversation factor for V_{Ca} in mV.

1.3 Synaptic dynamics

Synaptic currents were modelled in a manner similar to ionic currents as given by the following equation:

$$I_{syn} = \bar{g}_{syn} \cdot s(t) \cdot (V_m - E_{syn}) \tag{S83}$$

where $\bar{g}_{syn}$ is the maximal synaptic conductance, $s(t)$ is the synaptic conductance function and E_{syn} is the reversal potential of the synapse. The time-dependent conductance $s(t)$ can be modelled in several ways depending on the type of neurotransmitter-mediated synapse, as described below (Destexhe et al., 1998).

1.3.1 AMPA/GABA$_\mathrm{A}$-mediated synapse modelling

In the formalism by Destexhe et al. (Destexhe et al., 1994, 1998), postsynaptic currents mediated by glutamate AMPA and GABAergic GABA$_\mathrm{A}$ receptors are modelled using a gating variable $s(t)$ which denotes the fraction of open synaptic channels at time t. Open synaptic channels refer to postsynaptic receptors that neurotransmitter molecules bind to, followed by the opening of ion channels. In this way, the synaptic conductance function is described by:

$$\frac{ds}{dt} = \alpha[T](1 - s) - \beta s \tag{S84}$$

where $[T]$ represents the neurotransmitter concentration released into the synaptic cleft upon the arrival of a presynaptic spike, and α and β are rate constants describing the binding of neurotransmitter.

Upon the arrival of a presynaptic spike at $t = t_0$, $[T]$ jumps to a maximal concentration value C_{max}, and at $t = C_{dur}$ (the duration of neurotransmitter-mediated pulse), $[T]$ falls back to 0. After the neurotransmitter-mediated pulse is gone, $s(t)$ decays exponentially. Thus, solving Equation (S84), we get:

$$s(t) = \begin{cases} s_\infty + (s(t_0) - s_\infty)e^{-\frac{t}{\tau_s}}, & t_0 \leq t < C_{dur} \\ \left[s_\infty + (s(t_0) - s_\infty)e^{-\frac{C_{dur}}{\tau_s}}\right]e^{-\beta(t-C_{dur})}, & \\ & t \geq C_{dur} \end{cases} \tag{S85}$$

where

$$s_\infty = \frac{\alpha C_{max}}{\alpha C_{max} + \beta} \quad \text{and} \quad \tau_s = \frac{1}{\alpha C_{max} + \beta} \tag{S86}$$

For AMPA-mediated synapses, $E_{syn} = 0$ mV, $C_{max} = 0.5$ mM and $C_{dur} = 0.3$ ms, while α and β parameters vary according to the synapse type, as presented in Tables 3–4 from the main text. For GABA$_A$-mediated synapses, $E_{syn} = -85$ mV, $C_{max} = 0.5$ mM, and $C_{dur_{Control}} = 0.3$ ms, while values of $\alpha_{Control}$ and $\beta_{Control}$ vary according to the synapse type, as presented in Tables 3–4 from the main text. To model post-ALLO GABA$_A$-mediated synapses, $\alpha_{post-ALLO} = \alpha_{Control} \times 1.58$, $\beta_{post-ALLO} = \beta_{Control} \times 0.74$, and $C_{dur_{post-ALLO}} = C_{dur_{Control}} \times 1.14$.

1.3.2 GABA$_B$-mediated synapse modelling

The dynamics of GABA$_B$-mediated synapses followed the formalism by Destexhe et al. (Destexhe et al., 1998). GABA$_B$-mediated synapses active *indirectly*, following a series of intracellular events. In particular, the neurotransmitter binding event activates an intracellular complex called a G-protein which activates potassium channels ultimately hyperpolarizing and causing inhibition of the postsynaptic neuron. In this way, the synaptic conductance function is described by:

$$s(t) = \frac{g^4}{g^4 + K_d} \tag{S87}$$

$$\frac{dr}{dt} = K_1[T](1 - r) - K_2 r \tag{S88}$$

$$\frac{dg}{dt} = K_3 r - K_4 g \tag{S89}$$

where r is the fraction of GABA$_B$ receptors in the activated form, g is the normalized G-protein concentration in activated form given in μM, $K_d = 100\ \mu\text{M}^4$ is the dissociation constant of G-protein binding on potassium channels, $K_1 = 0.09$ (ms mM)$^{-1}$ is the forward binding rate to the receptor, $K_2 = 0.0012$ ms^{-1} is the unbinding rate, $K_3 = 0.18$ ms^{-1} is the rate of G-protein production, $K_4 = 0.034$ ms^{-1} is the rate of G-protein decay, and $E_{syn} = -95$ mV.

2 MODEL PARAMETERS

Table S2. Maximal conductance parameters (in mS/cm^2) for each neuron type.

g_{ion}	RS	IB	NRS	LTS	TC	RE
g_{Naf}	200	200	200	170	90	200
g_{Nap}	0.16	0.16	0.1	0.16	-	-
g_K	-	-	-	-	10	20
g_{Kdr}	170	170	170	100	-	-
g_{Ka}	75	17	20	1	-	-
g_{K2}	35	27	40	9	-	-
g_{Km}	43	40	55	35	-	-
g_{Kc}	20	20	15	15	-	-
g_{Kahp}	0.5	3.5	35	0.1	-	-
g_{CaT}	0.1	0.1	0.1	0.05	2	3
g_{CaL}	10	11	2	0.1	-	-
g_h	0.1	0.1	0.1	0.1	$\mathcal{N}(0.0175,\, (8 \times 10^{-7})^2)$	-
g_{Leak}	2	2	2	2.4	0.01	0.05
g_{KLeak}	-	-	-	-	$\mathcal{N}(4,\, (3 \times 10^{-4})^2)$	-

Table S3. The number of presynaptic neurons that each postsynaptic neuron connects to ($nPrePost$) in the 5-95 (nIB:nRS) model configuration.

$nPrePost$	Number of cells	$nPrePost$	Number of cells
nRSRS	11	nLTSRS	11
nRSIB	11	nLTSIB	11
nRSNRS	11	nLTSNRS	11
nRSLTS	10	nTCRE	11
nIBIB	11	nTCNRS	21
nIBRS	1	nTCIB	21
nIBNRS	1	nTCRS	21
nIBLTS	1	nTCLTS	21
nNRSNRS	11	nRERE	11
nNRSRS	9	nRETC	11
nNRSIB	11		
nNRSLTS	8		
nNRSTC	16		
nNRSRE	16		

3 EXTENDED METHODOLOGICAL DETAILS

3.1 Model fitting procedure

The model fitting procedure aimed to produce target behaviour qualitatively similar to the models upon which we have built this work (Destexhe, 1998; Traub et al., 2005). To assess model fitness, we defined specific evaluation metrics that characterize network behaviour in two critical states: healthy spindles and pathological spike-wave-discharges (SWDs). Our previous model guided the qualitative and quantitative criteria for both states (Ahmed and Campbell, 2024), the details of which are provided in (Ahmed, 2025).

Since the thalamus component was well-defined in (Destexhe, 1998), we kept it unchanged, including its synaptic weights and other related parameters. Similarly, all parameters associated with GABA_B synapses were preserved as defined in (Destexhe, 1998). Instead, we focused on fitting parameters corresponding to AMPA and GABA_A synapses within the cortex and between the cortex and thalamus. For each synapse, we sought the optimal set of parameters $\bar{g}_{syn}$, α, and β—as defined in Equations (8)–(10) from the main text—that would enable the network to display a default mode of spindle oscillations while maintaining

Table S4. The number of presynaptic neurons that each postsynaptic neuron connects to ($nPrePost$) in the 95-5 (nIB:nRS) model configuration.

$nPrePost$	Number of cells	$nPrePost$	Number of cells
nRSRS	11	nLTSRS	11
nRSIB	1	nLTSIB	11
nRSNRS	1	nLTSNRS	11
nRSLTS	1	nTCRE	11
nIBIB	11	nTCNRS	21
nIBRS	11	nTCIB	21
nIBNRS	11	nTCRS	21
nIBLTS	10	nTCLTS	21
nNRSNRS	11	nRERE	11
nNRSRS	11	nRETC	11
nNRSIB	9		
nNRSLTS	8		
nNRSTC	16		
nNRSRE	16		

the ability to transition to a spike-wave discharge (SWD) state under cortical disinhibition. This resulted in a 63-dimensional parameter search space. The choice of $\bar{g}_{syn}$, α, and β as fitting parameters was guided by prior optimization and dimensionality reduction efforts. Furthermore, the search space was restricted by narrowing each parameter to select values within particular ranges. Model fitting proceeded in multiple stages, beginning with the cortical layer 6-thalamus circuit, then adding cortical layer 5 cells, one population at a time. At each stage, synapses were incrementally added to the network, with the corresponding parameter triplet ($\bar{g}_{syn}$, α, β) optimized through a grid search to maintain target behaviour before introducing the next synaptic connection. Throughout, the model was periodically evaluated under two conditions—total cortical inhibition and total disinhibition—to ensure emergence of expected behaviours. Model outputs were assessed using an automated procedure with predefined criteria, supplemented by manual inspection of individual neuron firing patterns for physiological plausibility. We tested our model's robustness by exploring parameter clouds with 30–200% variation around each nominal triplet, with the variation range depending on synapse type and insights from the sequential fitting process. For synapses that strongly influenced network behaviour, we tested smaller neighbourhoods around the nominal values. Within these clouds, multiple triplets met our filtering criteria, indicating local robustness. Further details on the selection of fitting parameters and the staged fitting procedure can be found in (Ahmed, 2025).

3.2 Allopregnanolone application experiment curve fitting

Inhibitory postsynaptic currents mediated by $GABA_A$ receptors post application of allopregnanolone, obtained from experimental works (thereby referred to as I_{data}), were fit using the following equation:

$$I_{GABAa} = -\min(\tilde{I}_{data}) \cdot \frac{1}{s(C_{dur})} \cdot s(t) \quad \text{(S90)}$$

where

$$\tilde{I}_{data} = \frac{I_{data}}{(V_{hold} + 85)} \quad \text{(S91)}$$

$$s(t) = \begin{cases} \frac{\alpha C_{max}}{\alpha C_{max}+\beta}\left(1 - e^{-t(\alpha C_{max}+\beta)}\right), & 0 \leq t < C_{dur} \\ \left[\frac{\alpha C_{max}}{\alpha C_{max}+\beta}\left(1 - e^{-C_{dur}(\alpha C_{max}+\beta)}\right)\right] e^{-\beta(t-C_{dur})}, & t \geq C_{dur} \end{cases} \tag{S92}$$

All experimental data were scaled by factoring out the voltage dependent term for curve fitting purposes. This allowed for the isolation of the synaptic gating variable $s(t)$ dynamics, making the analysis of the synaptic gating more direct and interpretable. We interpret the scaling factor $\min(\tilde{I}_{data}) \cdot \frac{1}{s(C_{dur})}$ as the channel's maximal intrinsic conductance where $s(C_{dur})$ is equal to the maximum of the gating variable and $\min(\tilde{I}_{data})$ represents the maximal conductance.

We used MATLAB's *lsqcurvefit* function to find the best-fitting α, β and C_{dur} parameters in Equation (S90), for the given data. By pre-scaling the data, these parameters could be estimated to directly fit $s(t)$. As illustrated in Supplementary Figure S1, without pre-scaling, the relative importance of fitting different parts of the curve changes. This results in the optimization converging to different (non-ideal) local minima in the parameter space.

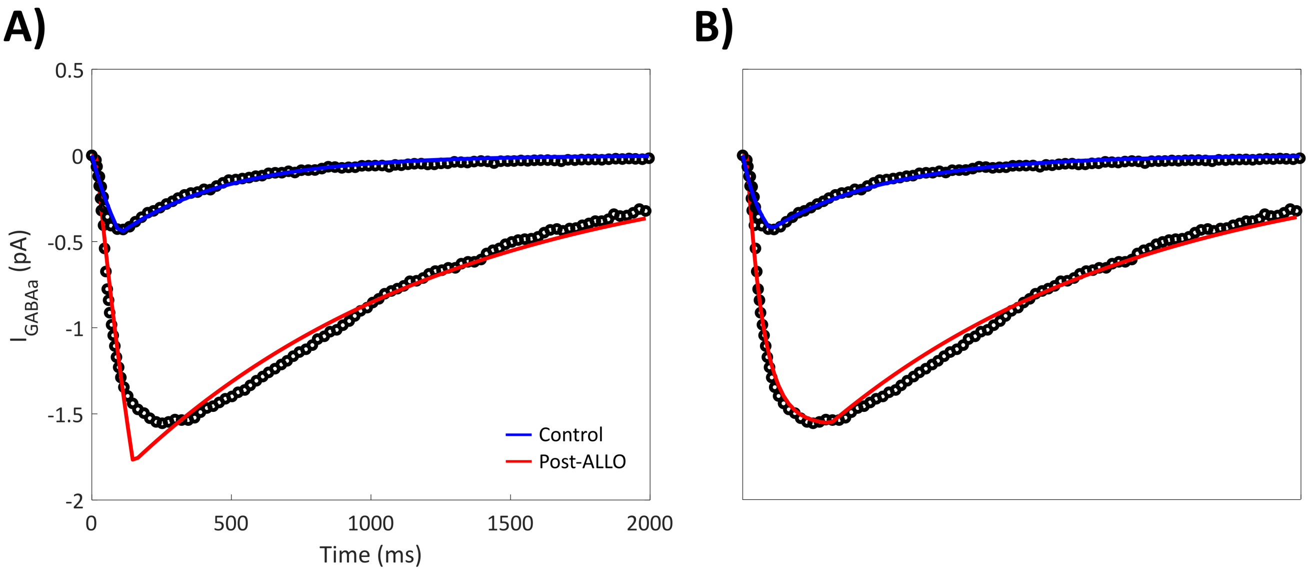

Figure S1. Sample curve fits of inhibitory postsynaptic currents post application of allopregnanolone, without (A) and with (B) pre-scaling data by factoring out the voltage dependent term.

3.3 Modelling increased frontocortical connectivity

REFERENCES

Ahmed, M. (2025). *Multi-scale Modelling of Neurosteroid-mediated Seizure Trajectories in Childhood Absence Epilepsy*. Ph.D. thesis, University of Waterloo

Ahmed, M. and Campbell, S. A. (2024). Modelling the effect of allopregnanolone on the resolution of spike-wave discharges. *Journal of Computational Neuroscience* 53, 115–130

Destexhe, A. (1998). Spike-and-wave oscillations based on the properties of GABAB receptors. *Journal of Neuroscience* 18, 9099–9111

Destexhe, A., Babloyantz, A., and Sejnowski, T. J. (1993). Ionic mechanisms for intrinsic slow oscillations in thalamic relay neurons. *Biophysical Journal* 65, 1538–1552

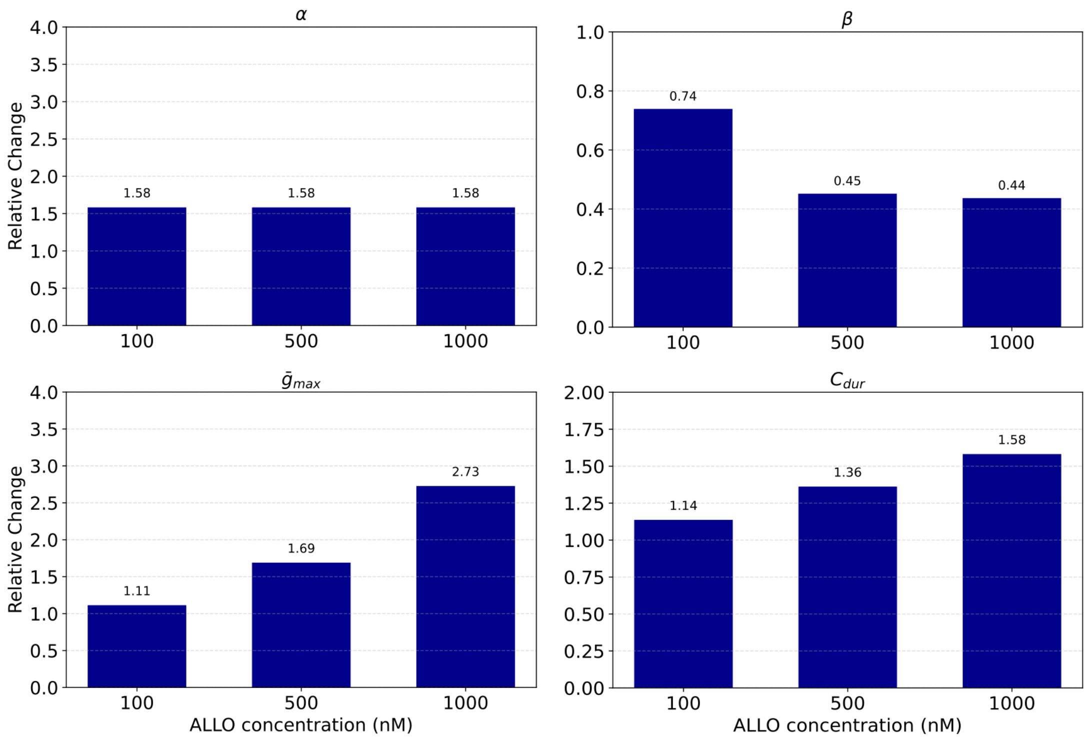

Figure S2. Comparison of the relative changes in $\bar{g}_{max}$, α, β and C_{dur} between control $GABA_A$ receptor activity and activity post-application of ALLO based on the fitting method described in Supplementary Section 3.2 to varying levels of ALLO reported in (Sullivan and Moenter, 2003).

Destexhe, A., Bal, T., McCormick, D. A., and Sejnowski, T. J. (1996a). Ionic mechanisms underlying synchronized oscillations and propagating waves in a model of ferret thalamic slices. *Journal of Neurophysiology* 76, 2049–2070

Destexhe, A., Contreras, D., Steriade, M., Sejnowski, T. J., and Huguenard, J. R. (1996b). In vivo, in vitro, and computational analysis of dendritic calcium currents in thalamic reticular neurons. *Journal of Neuroscience* 16, 169–185

Destexhe, A., Mainen, Z. F., and Sejnowski, T. J. (1994). Synthesis of models for excitable membranes, synaptic transmission and neuromodulation using a common kinetic formalism. *Journal of Computational Neuroscience* 1, 195–230

Destexhe, A., Mainen, Z. F., Sejnowski, T. J., et al. (1998). Kinetic models of synaptic transmission. *Methods in Neuronal Modeling* 2, 1–25

Huguenard, J. R. and McCormick, D. A. (1992). Simulation of the currents involved in rhythmic oscillations in thalamic relay neurons. *Journal of Neurophysiology* 68, 1373–1383

Martina, M. and Jonas, P. (1997). Functional differences in Na^+ channel gating between fast-spiking interneurones and principal neurones of rat hippocampus. *The Journal of Physiology* 505, 593

McCormick, D. A. and Huguenard, J. R. (1992). A model of the electrophysiological properties of thalamocortical relay neurons. *Journal of Neurophysiology* 68, 1384–1400

McCormick, D. A., Wang, Z., and Huguenard, J. (1993). Neurotransmitter control of neocortical neuronal activity and excitability. *Cerebral Cortex* 3, 387–398

Sullivan, S. D. and Moenter, S. M. (2003). Neurosteroids alter GABAergic postsynaptic currents in GnRH neurons: a possible mechanism for direct steroidal control. *Endocrinology* 144, 4366–4375

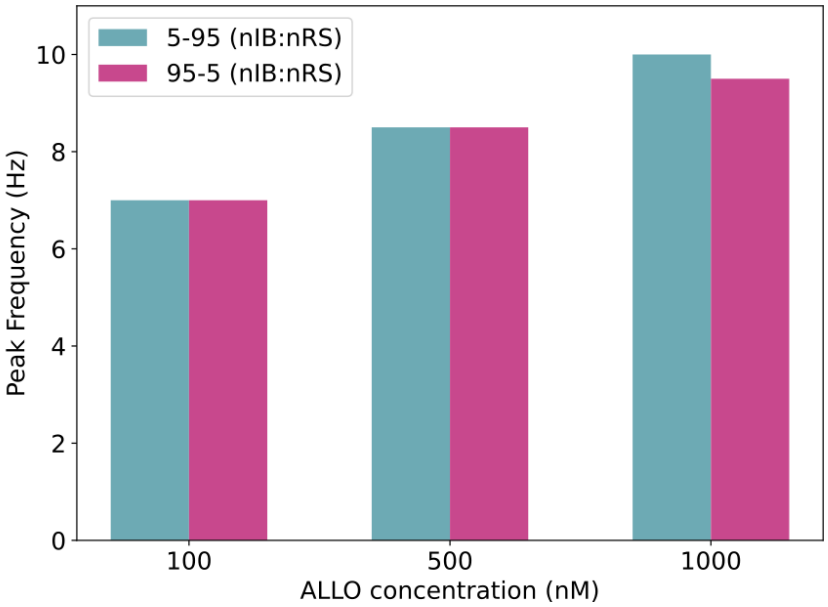

Figure S3. Comparison of network responses between the 5-95 and 95-5 (nIB:nRS) model configurations across physiological (100 nM) and supraphysiological (500 and 1000 nM) ALLO concentrations reported in (Sullivan and Moenter, 2003). At each ALLO concentration, networks were initialized with cortical disinhibition, and ALLO's effects were implemented using the synaptic and kinetic parameter values shown in Figure S2.

Traub, R. D., Buhl, E. H., Gloveli, T., and Whittington, M. A. (2003). Fast rhythmic bursting can be induced in layer 2/3 cortical neurons by enhancing persistent Na^+ conductance or by blocking BK channels. *Journal of Neurophysiology* 89, 909–921

Traub, R. D., Contreras, D., Cunningham, M. O., Murray, H., LeBeau, F. E. N., Roopun, A., et al. (2005). Single-column thalamocortical network model exhibiting gamma oscillations, sleep spindles, and epileptogenic bursts. *Journal of Neurophysiology* 93, 2194–2232

Traub, R. D., Jefferys, J. G., Miles, R., Whittington, M. A., and Tóth, K. (1994). A branching dendritic model of a rodent CA3 pyramidal neurone. *The Journal of Physiology* 481, 79–95

Traub, R. D., Wong, R. K., Miles, R., and Michelson, H. (1991). A model of a CA3 hippocampal pyramidal neuron incorporating voltage-clamp data on intrinsic conductances. *Journal of Neurophysiology* 66, 635–650

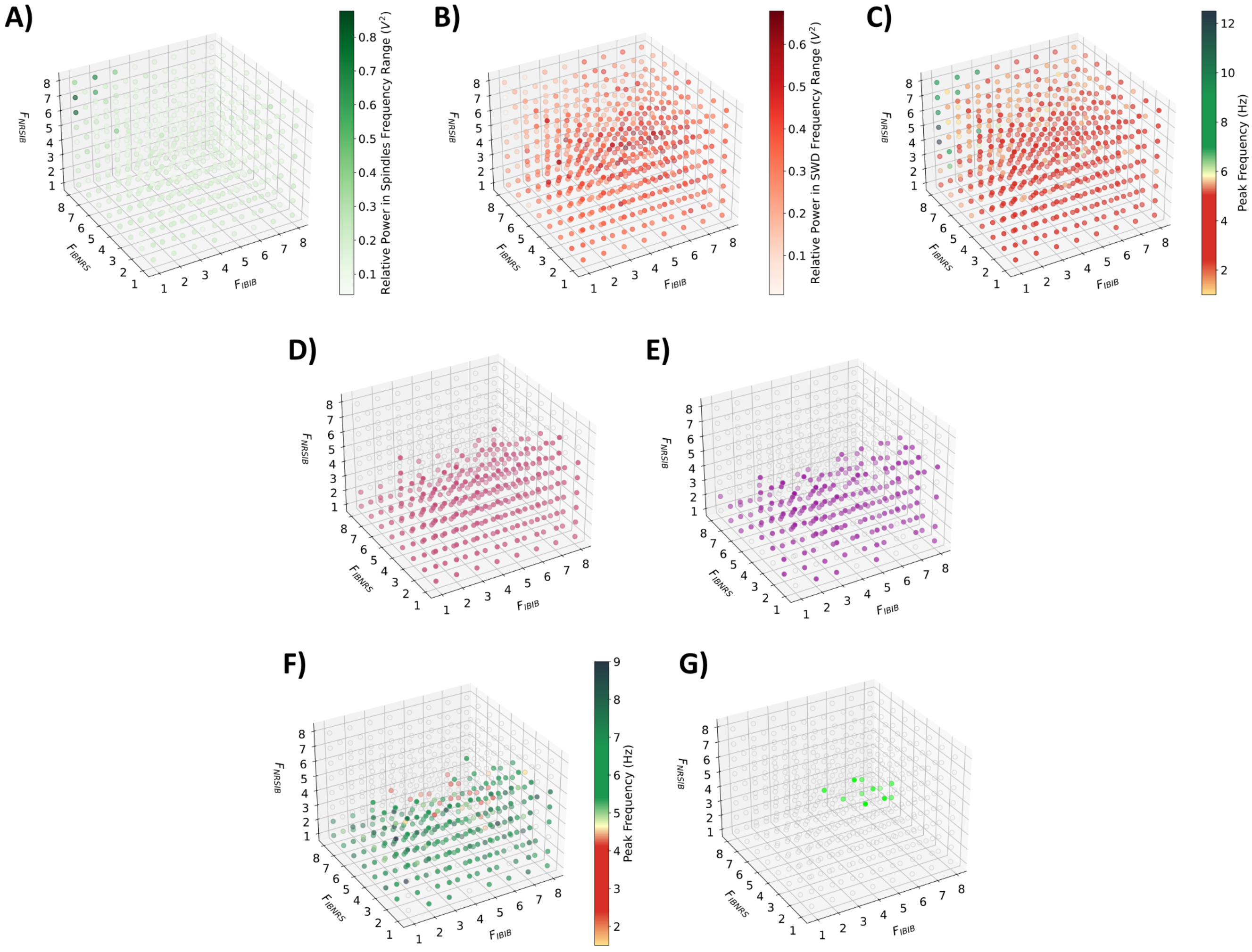

Figure S4. 3D visualization of the grid search results across $8^3 = 512$ parameter combinations within the $(F_{IBIB}, F_{IBNRS}, F_{NRSIB})$ parameter space using the 50-50 (nIB:nRS) model configuration. (A–C): Initial search using Control synapses with cortical $GABA_A$ conductance at 10% baseline, were filtered by examining the magnitude of: (A): relative power within spindles range, (B): relative power within SWDs range, and (C): peak frequency from power spectral density analysis. (D): Filtered parameter combinations from the initial search. (E): Parameter combinations that produce spindle-like oscillations when simulated with cortical $GABA_A$ conductance restored to normal (100% baseline) levels. (F): Parameter combinations that produce continued SWDs (in red) when the network is simulated using 10% baseline cortical $GABA_A$ conductance and post-ALLO synapses. (G): Final parameter set of $(F_{IBIB}, F_{IBNRS}, F_{NRSIB})$ for which the 50-50 (nIB:nRS) model exhibits continued SWDs post application of ALLO.
